# Changing biosynthesis of terpenoid percursors in rice through synthetic biology

**DOI:** 10.3389/fpls.2023.1133299

**Published:** 2023-07-03

**Authors:** Orio Basallo, Lucia Perez, Abel Lucido, Albert Sorribas, Alberto Marin-Saguino, Ester Vilaprinyo, Laura Perez-Fons, Alfonso Albacete, Cristina Martínez-Andújar, Paul D. Fraser, Paul Christou, Teresa Capell, Rui Alves

**Affiliations:** ^1^ Systems Biology Group, Department Ciències Mèdiques Bàsiques, Faculty of Medicine, Universitat de Lleida, Lleida, Spain; ^2^ Institut de Recerca Biomedica de Lleida (IRBLleida), Lleida, Spain; ^3^ Applied Plant Biotechnology Group, Department de Producció Vegetal I Ciència Florestal, Escola Tècnica Superior d'Enginyeria Agroalimentària i Forestal i de Veterinària (ETSEAFiV), Universitat de Lleida, Lleida, Spain; ^4^ Agrotecnio Centres de Recerca de Catalunya (CERCA) Center, Lleida, Spain; ^5^ School of Biological Sciences, Royal Holloway University of London, Egham Hill, United Kingdom; ^6^ Departament of Plant Nutrition, Center of Edaphology and Applied Biology of the Segura (CEBAS), Consejo Superior de Investigaciones Científicas (CSIC), Universidad de Murcia, Murcia, Spain; ^7^ Department of Plant Production and Agrotechnology, Institute for Agri-Food Research and Development of Murcia, Murcia, Spain; ^8^ ICREA, Catalan Institute for Research and Advanced Studies, Barcelona, Spain

**Keywords:** metabolic engineering, mathematical modelling, multi level modelling, MVA (mevalonic acid) pathway, MEP pathway, terpenoid synthetic biology

## Abstract

Many highly valued chemicals in the pharmaceutical, biotechnological, cosmetic, and biomedical industries belong to the terpenoid family. Biosynthesis of these chemicals relies on polymerization of Isopentenyl di-phosphate (IPP) and/or dimethylallyl diphosphate (DMAPP) monomers, which plants synthesize using two alternative pathways: a cytosolic mevalonic acid (MVA) pathway and a plastidic methyleritritol-4-phosphate (MEP) pathway. As such, developing plants for use as a platform to use IPP/DMAPP and produce high value terpenoids is an important biotechnological goal. Still, IPP/DMAPP are the precursors to many plant developmental hormones. This creates severe challenges in redirecting IPP/DMAPP towards production of non-cognate plant metabolites. A potential solution to this problem is increasing the IPP/DMAPP production flux *in planta*. Here, we aimed at discovering, understanding, and predicting the effects of increasing IPP/DMAPP production in plants through modelling. We used synthetic biology to create rice lines containing an additional ectopic MVA biosynthetic pathway for producing IPP/DMAPP. The rice lines express three alternative versions of the additional MVA pathway in the plastid, in addition to the normal endogenous pathways. We collected data for changes in macroscopic and molecular phenotypes, gene expression, isoprenoid content, and hormone abundance in those lines. To integrate the molecular and macroscopic data and develop a more in depth understanding of the effects of engineering the exogenous pathway in the mutant rice lines, we developed and analyzed data-centric, line-specific, multilevel mathematical models. These models connect the effects of variations in hormones and gene expression to changes in macroscopic plant phenotype and metabolite concentrations within the MVA and MEP pathways of WT and mutant rice lines. Our models allow us to predict how an exogenous IPP/DMAPP biosynthetic pathway affects the flux of terpenoid precursors. We also quantify the long-term effect of plant hormones on the dynamic behavior of IPP/DMAPP biosynthetic pathways in seeds, and predict plant characteristics, such as plant height, leaf size, and chlorophyll content from molecular data. In addition, our models are a tool that can be used in the future to help in prioritizing re-engineering strategies for the exogenous pathway in order to achieve specific metabolic goals.

## 1 Introduction

Terpenoids are a family of molecules with more than 22,000 different natural products (Harborne et al., 1991; Tetali, 2019; Zhou and Pichersky, 2020; Navale et al., 2021). Some family members have various crucial biological functions. For example, in plants, they work as hormones (gibberellin, abscisic acid, etc.), photosynthetic pigments (chlorophyll, phytol, carotenoids), electron carriers (ubiquinone, plastoquinone), mediators of the assembly of polysaccharides (polyprenyl phosphates) and structural components of membranes (phytosterols). They are also used for other purposes, such as antibiotics, herbivore repellents, toxins and pollinator attractants (Mcgarvey and Croteau, 1995).

**Figure 1:**
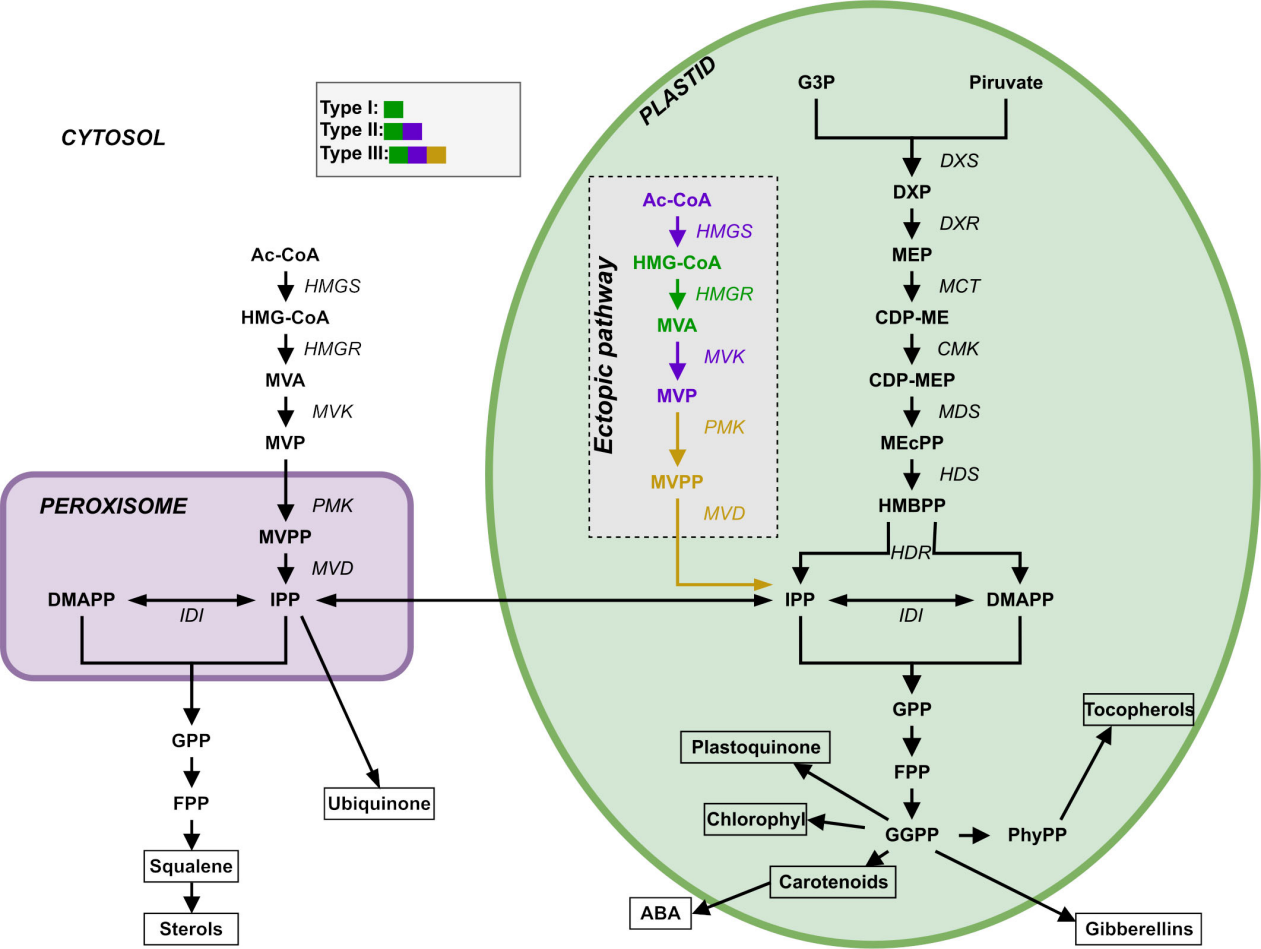
Representation of the two terpenoid biosynthesis pathways plus the ectopic pathway, the MVA pathway (left, cytosol and peroxisome) and the MEP pathway (right, plastid). DXR: DXP reductoisomerase; MCT: 2-C-methyl-D-erythrtle 4-phosphate cytidylyl transferase; CDP-ME: 4-(Citidine 5’-difosfo)-2-C-methyl-D-eritritol; CMK: 4-difosfocitidil-2-C-methyl-D-erythrtol kinase; CDP-MEP: 2-Fosfo-4-(cytidine 5’- diphospho)-2-C-methyl-D-eritritol; MDS: 2-C-methyl-D-eritritol 2,4-cyclodifosphate synthase; MEcPP: 2-C-methyl-D-eritritol 2,4-cycdiphosphate; HDS: 4-hydroxy-3-methylbut-2-en-1-il diphosphate synthase; HMBPP: 4-hydroxy-3-methylbut-2-in-1-il diphosphate; HDR: 4-hydroxy-3-methylbut-2-en-1-il diphosphate reductase; IDI: isopentenyl diphosphate Delta-isomerase; PhyPP: phytyl diphosphate.

Plants synthesize terpenoids from two metabolic precursors: Isopentenyl di-phosphate (IPP) and dimethylallyl diphosphate (DMAPP). Two compartmentally separated pathways synthesize these precursors (**Figure 1**). The mevalonic acid (MVA) pathway converts acetyl-CoA (Ac-CoA) to IPP and DMAPP. This pathway is mostly cytosolic, with a couple of reactions taking place in the peroxisome. The MVA pathway starts with the condensation of acetyl-CoA, a product of glycolysis, catalyzed by acetoacetyl-CoA thiolase and HMG-CoA synthase, followed by the conversion of HMG-CoA to mevalonate through HMG-CoA reductase. Mevalonate is subsequently phosphorylated and decarboxylated to yield IPP, which can be isomerized to DMAPP by the action of isopentenyl diphosphate isomerase (IDI). IPP and DMAPP are then used in the synthesis of phytosterols and ubiquinone (Mcgarvey and Croteau, 1995). The enzyme 3-hydroxy-3-methylglutaril-CoA reductase (HMGR) is a key enzyme in the regulation of the MVA pathway (Schaller et al., 1995). The second terpenoid-producing pathway is known as the methyleritritol-4-phosphate (MEP) pathway. This pathway is compartmentalized in plastids. In this pathway, glyceraldehyde 3-phosphate (G3P) and pyruvate derived from the Calvin cycle serve as the primary carbon sources for IPP and DMAPP production. The MEP pathway involves a series of enzymatic steps catalyzed by various enzymes, including 1-deoxy-D-xylulose 5-phosphate synthase (DXS), 1-deoxy-D-xylulose 5-phosphate reductoisomerase (DXR), and other downstream enzymes. It is responsible for the production of carotenoids, lateral chains of chlorophylls, plastoquinone, abscisic acid (ABA) and tocopherols (vitamin E, precursors and derivatives) (Eisenreich et al., 2001). Appendix S1 presents a more detailed description of both pathways.

While both pathways function independently, there is ample evidence of crosstalk between them (Hemmerlin et al., 2003; Hemmerlin, 2013). There is evidence for the exchange of some metabolic intermediates of the two pathways between compartments (Bick and Lange, 2003; Hemmerlin et al., 2003; Laule et al., 2003; Hemmerlin et al., 2006). The first intermediate of the MEP pathway, DXP, can diffuse between the plastid and the cytoplasm (Hemmerlin et al., 2003; Page et al., 2004; Lange et al., 2015). At the level of IPP and DMAPP, this exchange was measured to occur mainly in the plastid-to-cytoplasm direction, promoted by a one-way symport system (Bick and Lange, 2003; Dudareva et al., 2005). The direction of this metabolic exchange between cellular compartments may depend on physiological state and species. There is lack of convincing evidence that other intermediates of both pathways can diffuse between the two compartments (Hemmerlin, 2013). Hemmerlin et al. (2012) made an extensive review of the literature covering the metabolic pathways themselves (networks, regulation, biological advantage of having two separated pathways, etc.), crosstalk between pathways and the potential of terpenoid biosynthesis in bioengineered plants in biotechnology.

In recent years, synthetic biology has emerged as a powerful tool for engineering production of isoprenoids, mostly into *Saccharomyces cerevisiae* and other microbial hosts (Ro et al., 2006; Ajikumar et al., 2010; Jiang et al., 2017; Kang et al., 2017; Cravens et al., 2019; Luo et al., 2019; Gülck et al., 2020; Srinivasan and Smolke, 2020; Liew et al., 2022; Zhang et al., 2022). While microbial production is very attractive due to the speed and easiness of genetically manipulating microorganisms, plant production should remain center stage if we are to progress to a circular bio-economy (Shih, 2018). Some of the earliest examples include the modifications made to maize (Naqvi et al., 2009) and rice (Ye et al., 2000), in order to increase their vitamin content. In general, modulating metabolite levels in plants is attempted through varying combinations of the following strategies: increasing enzymatic activity, increasing availability of upstream precursors, blocking leakage of the compound by gene silencing, or inducing metabolite storage in a compartment (Zorrilla-López et al., 2013; Kotopka et al., 2018; Maeda, 2019; Zhu et al., 2021; Yang et al., 2022). Examples of plant modification to enhance production of many complex isoprenoids became increasingly common (reviewed in Liao et al. (2016)). For example, Kumar et al. modified the chloroplast genome of tobacco leaves in a non-transmissible way to code an exogenous MVA pathway (Kumar et al., 2012). A more recent example is the enhancement in production of sesquiterpene precursor FPP of the MEP pathway in tomato fruit (Chen et al., 2023). As such, plants are a coveted target for engineering pathways that produce high value terpenoid chemicals (Fuentes et al., 2016; Georgiev et al., 2018; Gülck et al., 2020; Grzech et al., 2023).

Pérez et al. (2022) used synthetic biology to create rice plants that have a stable and transmissible ectopic plastidial MVA pathway that coexists with the native MVA and MEP pathways and is expressed in endosperm. The goal of that study was to circumvent the regulation of the native MVA pathway and test the possibility of producing an excess of terpenoids precursors that could feed the biosynthesis of highly valuable terpenoids. They introduced three different combinations of exogenous WR1, HMGS, HMGR, MVK, PMK and MVD (**Figure 1**) genes encoding plastid-targeted enzymes, collecting transcriptomic, metabolic, and phenotypic data for the resulting mutant lines. The WR1 gene is a transcription factor that induces the expression of genes related to plastid glycolysis and fatty acid biosynthesis. HMGS codes for hydroxymethylglutaryl-CoA synthase, HMGR codes for a 3-hydroxy-3-methylglutaryl-coenzyme A reductase, MVK codes for a mevalonate kinase, PMK codes for a phosphomevalonate kinase, and MVD codes for a diphosphomevalonate decarboxylase.

Here, we further study the effect that adding this pathway has on rice by creating and characterizing new rice lines with alternative versions of the exogenous MVA pathway. Subsequently, we use multilevel mathematical modeling to integrate the data of all mutant lines, and predict the effect of genome modification on the concentrations of metabolic intermediates and on the fluxes going through the MEP, MVA, and ectopic plastidial MVA pathways. We also quantify the long-term effect of plant hormones on the dynamic behavior of IPP/DMAPP biosynthetic pathways in seeds, and predict plant characteristics, such as plant height, leaf size, and chlorophyll content from molecular data. In addition, our models are a tool that can be used in the future to help in prioritizing re-engineering strategies for the exogenous pathway in order to achieve specific metabolic goals.

## 2 Materials and methods

### 2.1 WT and mutant rice lines

We created three types of mutant rice lines using the procedures described in Pérez et al. (2022). Mutant Type I had exogenous HMGR; Mutant Type II had exogenous HMGS, HMGR and MVK; and Mutant Type III had exogenous HMGS, HMGR, MVK, PMK and MVD. We placed these six transgenes (*BjHMGS*, *tHMGR*, *CrMK*, *CrPMK*, and *CrMVD*) in three independent expression cassettes driven by endosperm-specific promoters. A transit peptide inserted at the beginning of the five enzymes in the MVA pathway directs them to the plastid. See Pérez et al. (2022) and **Supplementary Appendix S1** for the full details.

To create the mutant lines, we bombarded seven-day-old mature zygotic rice embryos (*Oryza sativa* cv. EYI105) with gold particles coated with the transformation vectors. We recovered transgenic plantlets and regenerated and hardened them off in soil. Genomic DNA was isolated from the callus and leaves of regenerated plants to confirm presence of the *BjHMGS*, *tHMGR*, *CrMK*, *CrPMK*, *CrMVD* and *OsWR1* through PCR (Pérez et al., 2022). We recovered 12 independent type I mutant lines, 10 independent type II mutant lines, and 12 independent mutant lines.

### 2.2 Hormone determination, gene expression, and plant phenotypes

We analyzed all rice lines after 12 weeks of growing in soil.

For each line we measured chlorophyll levels and analyzed the cytokinins trans-zeatin, zeatin riboside and isopentenyl adenine (iP); the gibberellins GA1, GA3 and GA4; the auxin indole-3-acetic acid (IAA); ABA; salicylic acid; jasmonic acid; and the ethylene precursor 1-aminocyclopropane-1-carboxylic acid (ACC) as described in Pérez et al. (2022) and **Supplementary Appendix S1**.

We also measured gene expression for *BjHMGS*, *tHMGR*, *CrMK*, *CrPMK*, *CrMVD*, and the endogenous MVA and MEP pathway genes *OsHMGS*, *OsHMGR*, *OsMK*, *OsPMK*, *OsMVD*, *OsDXS*, *OsDXR*, *OsMCT*, *OsCMK*, *OsMDS*, *OsHDS*, *OsHDR* and *OsIPPI* using qRT-PCR as described in Appendix S1.

We counted the number of leaves, measured the height of the plants from the base of the stem to the maximum extension of the flag leaf, and measured the length and maximum width of the last expanded leaf as described in Pérez et al. (2022). We used a SPAD meter at six points on the last expanded leaf to quantify leaf chlorophyll. We multiplied length and maximum width of the last expanded leaf by a correction factor of 0.75 to estimate leaf area.

All experimental measurements are provided in **Supplementary Data S1** and described in the results section of **Appendix S1**.

### 2.3 Mathematical modeling formalism

We used ordinary differential equation systems to model the biosynthesis of IPP/DMAPP. The mathematical formalism used to describe the flux dynamics is the saturating formalism (Sorribas et al., 2007; Alves et al., 2008). This formalism allows us to approximate the kinetics of any given reaction using a rational expression, where parameters have physical interpretations that are analogous to those found in classical enzyme kinetics rate expressions. In this formalism, we approximate the rate of a reaction in an inverse space at an operating point by:


(1) $$v\approx \frac{V\prod_{i=1}^{m}x_{i}^{\;}}{\prod_{i=1}^{m}\left(K_{i}+x_{i}^{\;}\right)+\prod_{b=1}^{p}\left(x_{b}^{\;}+K_{b}\right)}$$

V parameters represent apparent saturation rate constants for the reactions. Ki parameters represent apparent binding constants for the substrate(s) or inhibitor(s) of the reaction. While no activators were considered in our model, these can also be included using this formalism.

### 2.4 The endogenous MVA and MEP pathways

We modelled the wild type IPP and DMAPP production (i.e., the endogenous MVA and MEP pathways), using the canonical reaction set for each pathway, shown in **Figure 1**. We modelled the kinetics of each process, as well as those for the exchange fluxes of IPP and DMAPP between cytoplasm and plastid, using the rate expressions in **Supplementary Tables S1**–**S3**. We assume that the organism is able to maintain homeostasis of Acetyl-CoA and Acetoacetyl-CoA.

### 2.5 The ectopic MVA pathway in plastid

To model type I mutants, we added the reaction that transforms HMG-CoA_pl_ into MVA_pl_ to the plastid, as well as the cytoplasm-plastid exchange reactions for these two metabolites (**Supplementary Tables S3**–**S4**).

We extended the model for type I mutants to create the model for type II mutants. We included the reactions catalyzed by HMGS and MVK to the plastid compartment (**Supplementary Table S4**). Corresponding compartment exchange reactions for the substrates and products of these enzymes are also added (**Supplementary Table S3**).

We extended the model for type II mutants to create the model for type III mutants. We added the metabolite MVPP to the plastid by including the reaction that synthesize it. We also added the reaction that transforms MVPP_pl_ into IPP_pl_, (**Supplementary Table S4**). The plastid-cytoplasm exchange flux of MVPP_pl_ is included as well (**Supplementary Table S3**).

### 2.6 Exchange of MVA and MEP pathway metabolites between the cytoplasm and the plastid

Under physiological conditions, IPP and DMAPP mostly flow from the plastid into the cytosol (Bick and Lange, 2003). We implemented this observation by assuming that metabolites flow from the plastid to the cytosol at ten times the rate of the import reaction from the cytosol. Bick and Lange (Bick and Lange, 2003) also reported that other pathway intermediates were not actively transported between the two compartments. Other studies confirm this observation (for example, Wright et al. (2014)). However, those same studies show that in mutants overexpressing DXS, there is a second pool of MEcPP outside the chloroplast. In addition early intermediates of the MVA pathway can be found in the plastid space (Schneider et al., 1977).

Introducing an MVA pathway into the plastid as we did, may cause changes in the flux of MVA intermediates between the plastid and the cytoplasm. As such, we allowed for the possibility that HMG-CoA, MVA, MVP and MVPP enter and leave the plastid, albeit at very slow rates. **Supplementary Table S3** summarizes all reactions of material interchanged between plastid and cytosol.

### 2.7 Assembling the ordinary differential equation models for each type of rice

Each metabolite has its own differential equation in the model. The kinetic rate function, $f_{j}$ , for each process that produces a metabolite M appears as a positive term in the differential equation that determines the dynamic behavior of that metabolite. Similarly, the kinetic rate function, $f_{k}$ , for each process that consumes a metabolite appears as a negative term in the differential equation that determines the dynamic behavior of that metabolite:


(2) $$\frac{dM_{i}}{dt}=\sum_{j}f_{j}-\sum_{k}f_{k}$$

For the wild type and for each mutant type we assemble a type-specific system of ordinary differential equations (ODEs) that describes the dynamic behavior of all metabolites in the system. As such, we have four different ODE model types. These four models describe the dynamic behavior of all metabolites in the system in a type-specific manner.

### 2.8 Estimating rate constants, metabolite concentrations and variations in enzyme activity for each line

**Supplementary Tables S5** presents the basal kinetic constants for each reaction in the four model types. **Supplementary Table S6** presents the concentrations for the independent variables of the four model types.

To personalize the models and make them line specific we need to weigh the rate constants of the relevant type-specific model by the variations in enzyme activity of the individual line of interest. To do so we searched the literature for information about the correlation between changes in gene expression and enzyme activities in the MEP and MVA pathways. As we found no such information, we modeled variations in the enzyme activities of the mutant lines as described in Comas et al. (2016): changes in gene expression with respect to the WT are assumed to be proportional to changes in protein activity. This is the simplest possible assumption about the relationship between changes in gene expression and changes in enzyme activity.

We implement this assumption in the models by explicitly considering the enzymes that catalyze each reaction in the rate expressions. As $V_{max}\approx k_{cat}Enzyme$ , the model for the WT sets the enzyme activity to be 1 (the basal state). As we model mutant lines, we assume that changes in gene expression are proportional to changes in enzyme activity. Thus,


(3) $$Enzyme_{line\;i}=Enzyme_{wild\;type}\times \left(Gene\;expression_{line\;i}/Gene\;expression_{wild\;type}\right)$$

In the end we obtained one line-specific model for the wild type rice, 12 line specific models for type I mutant lines, 10 line specific models for type II mutants, and 12 line specific models for type III mutants.

### 2.9 Stability analysis

While many biological phenomena are rhythmic, overall, biological systems survive because they can achieve homeostasis (Wang et al., 2022). In other words, metabolism remains buffered and stable against normal environmental fluctuations. This occurs for the MEP pathway (Wright et al., 2014). Mathematically this situation is described by a stable steady state. It is well known that, when modeling homeostatic situations, lack of stability is a good diagnostic tool for model incompleteness (Savageau, 1975; Grimbs et al., 2007; Schmidt et al., 2011; Voit, 2013). For example, Ni and Savageau (1996a; 1996b) used this type of diagnostic tool to predict regulatory interactions that could stabilize a model of the red blood cell metabolism. Because of this, we perform stability analysis of the line-specific rice models in order to identify possible model improvements that can stabilize unstable steady states.

An efficient way to assess stability is by calculating the eigenvalues of the Jacobian matrix of the ODE system, which are complex numbers (Voit, 2013). If the real parts of all eigenvalues are negative, the system is stable. Otherwise, the system is unstable. The Jacobian matrix is constructed by taking the partially derivatives of the right-hand side of the ODEs ( $f_{i}$ ) with respect to each state variable ( ${\text{x}}_{j}$ ), as shown in Eq. 4.


(4) $$J=D_{x}f=f_{x}=\frac{\partial f_{i}}{\partial x_{j}}=\left(\begin{array}{cc} \begin{array}{cc} \frac{\partial f_{1}}{\partial x_{1}} & \frac{\partial f_{1}}{\partial x_{2}}\\ \frac{\partial f_{2}}{\partial x_{1}} & \frac{\partial f_{2}}{\partial x_{2}} \end{array} & \begin{array}{cc} \ldots & \frac{\partial f_{1}}{\partial x_{n}}\\ \ldots & \frac{\partial f_{2}}{\partial x_{n}} \end{array}\\ \begin{array}{cc} \vdots & \vdots \\ \frac{\partial f_{n}}{\partial x_{1}} & \frac{\partial f_{n}}{\partial x_{2}} \end{array} & \begin{array}{cc} \ddots & \vdots \\ \ldots & \frac{\partial f_{n}}{\partial x_{n}} \end{array} \end{array}\right)$$

### 2.10 Sensitivity analysis

In addition to steady state stability, another tool for model diagnostic is steady state robustness. Reasonable models generate steady states that are robust, and have low sensitivity to parameter changes (Savageau, 1975; Voit, 2013). Sensitivity measures how much a dependent variable or output changes when a parameter is altered (Comas et al., 2016). Parameters with high sensitivities tend to identify where information may be incomplete or inaccurate.

As such, we performed a sensitivity analysis to identify which steps of the pathway could have additional regulation that we were ignoring. We calculated logarithmic, or relative, steady-state parameter sensitivities, which measure the “relative change in a system variable (X) that is caused by a relative change in a parameter (p)” (Voit, 1991):


(5) $$\bar{S}\left(X,p\right)=\frac{\partial X/X}{\partial p/p}=\frac{\partial \log X}{\partial \log p}$$

This sensitivity analysis generates a matrix of sensitivities for each line. Each element of the matrix (of dimensions m×n ) represents the sensitivity $S_{i,j}$ of metabolite $M_{i}$ to parameter $p_{j}$ . To facilitate visualization of sensitivity analysis results and comparison between lines, we compressed the sensitivity analysis matrices for each line in two ways.

First, to see how much a line is sensitive to a certain parameter over all metabolites (variables of the system) we calculate the following index (Comas et al., 2016):


(6) $$S{'}_{j}=\frac{\sqrt{\sum_{i=1}^{n}S_{i,j}^{2}}}{\sqrt{n}}$$

In other words, we calculate the size (or Euclidean norm) of the vector whose components are the sensitivity of each metabolite to parameter $p_{j}$ , normalized by the number of metabolites in each mutant line (for example, models for Type I lines have 16 metabolites, while those for Type III have 18). We use the Euclidean norm of the sensitivity vectors as a way to represent aggregate sensitivities with a single metric to facilitate visual representation and analysis. Further, and because the number of metabolites increases from type I to type II and from type II to type III models, we make $S{'}_{j}$ comparable between models by normalizing it by the number of metabolites considered in the model. Second, to see how sensitive a metabolite is to all parameters in a line we calculate the following index:


(7) $$S{'}_{i}=\frac{\sqrt{\sum_{j=1}^{m}S_{i,j}^{2}}}{\sqrt{m}}$$

As with Eq 6, we calculate the size (or Euclidean norm) of the vector whose components are the sensitivity of the same metabolite $\;M_{i}$ to each parameter $p_{j}$ , normalized by the number of parameters in each mutant line. We use the Euclidean norm of the sensitivity vectors as a way to represent aggregate sensitivities with a single metric to facilitate visual representation and analysis. Further, and because the number of parameters increases from type I to type II and from type II to type III models, we make $S{'}_{j}$ comparable between models by normalizing it by the number of parameters considered in the model.

### 2.11 Investigating hormone influence

To investigate if we could use plant hormone levels as predictors of dynamic behavior in IPP/DMAPP biosynthesis in seeds we performed correlation analysis between hormones and metabolites, as well as genes, as described in Section 1.9 of Appendix S1. Significant effects were then included in the ODE models using one of two possible formalisms:


(8) $$M_{i}=\alpha H_{j}^{g_{ij}},$$

(9) $$M_{i}=\alpha {\left(\frac{H_{j}}{K+H_{j}}\right)}^{g_{ij}}$$

where $M_{i}$ is the concentration of metabolite *i* in the model, H_j_ is the level of hormone *j* at twelve weeks, and α and g are constants. We chose between the two alternatives in the following way. First we adjust a linear model of Log[ $M_{i}$ ] as a function of Log[ $H_{j}]$ . A combination of low adjusted $R^{2}$ and high $\left|g_{ij}\right|$ suggests a potentially strong influence of the hormone levels on metabolite concentrations (high d $\left|g_{ij}\right|$ ) over a small range of hormone levels (low adjusted $R^{2}$ ). In this situation, we assumed a saturation effect and used Eq 9 to model hormone influence on metabolite production and consumption. Otherwise, we used Eq 8, as it uses a smaller number of parameters and minimizes the possibility of overfitting the model to the data. The threshold for selecting the one or the other formalism was set at 0.5 for the ratio $\left|R_{adj}^{2}/g\right|.$ If $\left|R_{adj}^{2}/g\right|>0.5$ we use the power law formalism. The right-hand side of the equations modify the ODEs by multiplying the terms that involve production/consumption of the metabolite and involving those enzymes whose genes levels also correlate to hormone levels, in a way that makes the observed correlations affect the production and consumption rates of the metabolites.

For a more detailed procedure, see the **Supplementary Appendix S1**.

We note that, when hormone levels were below the experimental detection threshold, we reverted the kinetic expression presented in **Supplementary Table S7** to the original model, using a piece-wise approximation to solve the differential equations.

### 2.12 Phenotype models

We used a form of forward stepwise regression (Efroymson, 1960) to investigate how the different phenotypic variables might be predicted from hormones levels, gene expression and metabolite concentrations. We analyzed the following plant phenotypic characteristics: *Height*, number of *Leaves*, *Leaf Length*, *Leaf Width*, and *Chlorophyl* levels. We split experimental data according to mutant type, so that the analysis and model building was performed three times, one for each mutant type. We investigate phenotype as a function of the predictor variables gene expression, hormone levels, and metabolite concentrations.

The first step of the regression analysis was building independent linear models with one predictor variable.

The second step of the regression analysis was to select the predictor variables that had a significant ( α=0.05 ) effect on the phenotype and whose model had an adjusted R^2^ greater than 0.2. If only one model has a significant effect, we would choose that one. If more than one predictor variable has a significant effect in explaining the predicted variable, we chose the model for the variable with the highest adjusted R^2^. If the adjusted R^2^ is similar between models, we chose the model with the lowest AICc (AIC corrected for small sample sizes) score. The lower this score, the lower the chance that a model over fits the observations. If the AICc is similar for more than one model, we selected the predictor variable with the highest adjusted R^2^. At this stage, we have a one variable model.

The third step of the analysis was to create models with all possible combinations of predictor variables where one of the elements of the pair is the predictor variable selected in step one. We then selected the best two variable models as described in the previous paragraph, while making sure they are not collinear.

We repeated steps two and three and stopped when adding a new variable did not improve the explanatory power of the model. Thus, for a given set of significant predictors $\left\{x_{1},x_{2},\;\ldots ,x_{n}\right\}$ , the model would be:


(10) $$\hat{y}=\beta_{0}+\beta_{1}x_{1}+\beta_{2}x_{2}+\ldots +\beta_{n}x_{n}$$

We used forward stepwise regression instead of the more traditional multilinear modelling approach that starts from Eq. 10 and eliminates all variables that have no effect because the number of data points is smaller than the number of parameters to fit to the full multilinear model.

## 3 Results

### 3.1 Mathematical description of IPP and DMAPP biosynthetic pathways

The full mathematical description of IPP/DMAPP biosynthesis consists of 14, 16, 17 and 18 differential equations for the wild type (WT), Mutant Type I (MT-I), Mutant Type II (MT-II) and Mutant Type III (MT-III), respectively. Eq 11 shows the overall ODE systems for the four model types:


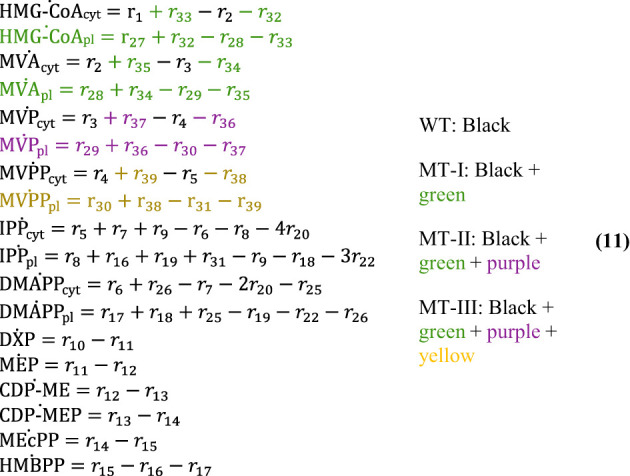


### 3.2 MVA and MEP homeostasis is robust in the WT line

**Table 1** provides the concentrations of each metabolite estimated from the model in the WT line. The system can achieve homeostasis (stable steady state in mathematical nomenclature). Stable steady states have negative real parts for the eigenvalues of the system’s Jacobean matrix (**Table 2**). The model also estimates that pathway substrates (HMG-CoA and DXP) and end-products (DMAPP and IPP) concentrations are, in general, larger than those of intermediate metabolites, which is another hallmark of a well-behaved biosynthetic pathway (Alves and Savageau, 2000).

**Table 1:** Concentrations of the basal model.

Metabolites	[mM]
HMGCoA_cyt_	0.983
MVA_cyt_	3.5x10^-5^
MVP_cyt_	3.98x10^-4^
MVPP_cyt_	3.36x10^-5^
IPP_cyt_	0.109
IPP_pla_	0.0801
DMAPP_cyt_	0.136
DMAPP_pla_	0.124
DXP	0.0133
MEP	1.15x10^-3^
CDPME	1.11x10^-4^
CDPMEP	0.0920
MECPP	0.657
HMBPP	3.52x10^-4^

**Table 2:** Eigenvalues for the Steady State.

	Real	Im
Eigenvalue1	-928.693	0
Eigenvalue2	-891.953	0
Eigenvalue3	-872.862	0
Eigenvalue4	-272.249	0
Eigenvalue5	-83.721	0
Eigenvalue6	-78.084	0
Eigenvalue7	-16.974	0
Eigenvalue8	-11.845	0
Eigenvalue9	-6.668	0
Eigenvalue10	-2.011	0
Eigenvalue11	-0.913	0
Eigenvalue12	-0.545	0
Eigenvalue13	-0.110	0
Eigenvalue14	-0.031	0

To understand how perturbations in parameters may affect the ability of the system to maintain homeostasis, we calculated the logarithmic sensitivity of the steady state Jacobian eigenvalues to each parameter of the model (**Supplementary Table S8**). The model has over eighty parameters and eigenvalues have sensitivities that are above one (in absolute values) to thirty of them. The parameters to which more eigenvalues are sensitive concentrate in reactions r2 (HMG-CoA_cyt_ → MVA_cyt_), r3 (MVA_cyt_ → MVP_cyt_), r4 (MVP_cyt_ → MVPP_cyt_), and r6 (IPP_cyt_ → DMAPP_cyt_) of the MVA pathway and reactions r10 (Glyceraldehyde-3-P + Pyruvate → DXP) and r18 (IPP_pl_ → DMAPP_pl_) of the MEP pathways. This suggests that modifying isoprenoid biosynthesis could destabilize the physiological steady states of the plant.

### 3.3 Homeostatic concentrations are robust to enzyme mutations in the WT line

Sensitivity analysis identifies the parameters to which the various variables of the model are most sensitive, as described in (Sorribas et al., 2007; Alves et al., 2008). A high sensitivity of a variable to a parameter indicates that small changes in that parameter might lead to big changes in the variable.

Plausible models of biological systems have low sensitivities to most parameters (Savageau, 1976; Kitano, 2007). The logarithmic sensitivity analysis of the dependent concentrations of the WT model with respect to each parameter of the model we performed shows that our model fits this quality criterion. Only 51 out of 728 sensitivities are larger than one (**Supplementary Table S9**). DMAPP and IPP are the metabolites with the highest sensitivities. High sensitivities are well known to identify the parts of a system that need to be modeled in more detail when additional information becomes available (Savageau, 1976; Kitano, 2007). This is consistent with the fact that we modeled IPP and DMAPP usage only through simple sink reactions, without considering any metabolic and regulatory details.

### 3.4 Existence of homeostatic behavior in the mutant lines requires posttranscriptional regulation of protein activity

We implement the models for the biosynthetic pathways in each mutant line using the same procedure as that for the WT line (sections 2.3 to 2.6). These systems do not reach homeostasis, having unstable steady states with a few intermediate metabolites accumulating over time. This suggests that the models are not plausible representations of the biological situation (Savageau, 1971; Savageau, 1975; Savageau, 1976; Voit, 2013).

Biological systems can stabilize steady states and reach homeostasis by adjusting the activity of enzymes in a pathway, for example through post-transcriptional regulation of protein levels and activity. We investigated whether emulating this type of adjustment would stabilize the steady states in the models.

First, we identified the metabolites that accumulated in each line, which were DXP, CDP-MEP, MEcPP, or combinations thereof. Reactions r11, r14, and r15 of **Table S2** either produce or consume these metabolites. Using a minimal intervention policy, we scanned the values for the Vmax parameters of reactions Vmax9, Vmax12 and Vmax13 in order to identify the minimum change in those parameters that would stabilize the steady state of each mutant line.

To stabilize homeostasis in the models we scanned one-dimensional, two-dimensional and three-dimensional parameter spaces and found the values of Vmax that stabilized the steady state in each model. The sets of Vmax that made the model for each mutant line stable were stored in a candidate sets list and we chose the final parameter set as the one with minimum normalized Euclidian distance to the original set of parameter values for that line. We provide the list of stabilized parameter values for each line in **Supplementary Data S1** – Model_stabilization. **Supplementary Figure S1** shows that Type II and Type III mutants require larger changes in parameter values than Type I mutants.

### 3.5 Stabilized homeostatic concentrations are robust to enzyme mutations in the mutant lines

**Figure 2:**
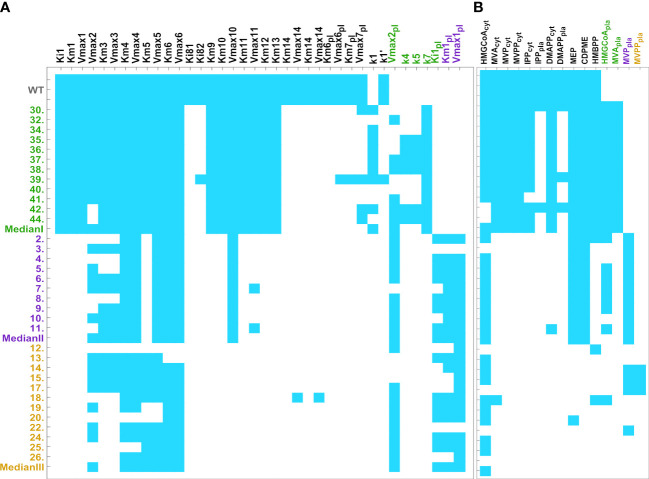
Heatmap of pooled sensitivities by parameter (left, threshold = 0.059) and by metabolite (right, threshold = 0.025), compared across mutant lines. Endogenous pathways are equal in all mutant types and have the same reactions. The models for the exogenous MVA plastid pathway are mutant type specific. Type III mutants include the reactions catalyzed by the five exogenous genes, HMGS, MVK, HMGR, PMK, and MVD. Type II mutants include the reactions catalyzed by HMGS, HMGR, and MVK. Type I mutants only include the reaction catalyzed by exogenous HMGR. As such, type III models have more kinetic parameters than type II. Type I has the lowest number of parameters. **(A)** Aggregate sensitivity of all metabolites to each parameter. **(B)** Aggregate sensitivity of each metabolite to all parameters.

We performed a sensitivity analysis of the stable homeostatic concentrations with respect to each enzyme parameter, in each mutant line (**Figure 2A**). We find that those concentrations are very robust, with 3% of all individual sensitivities being higher than 1 in absolute value in Type I mutants. This number goes down to 1.8% in Type II mutants and 1.4% in Type III mutants. The total number of individual sensitivities calculated for each line is higher than 900.

Globally, we found that, for each parameter $p_{j}$ , the aggregated sensitivity $S{'}_{j}\;$ of all metabolites to that parameter decreases in mutant lines with respect to the WT. The global sensitivities to each parameter decrease in the following order: WT>Type I lines> Type II lines> Type III lines. We also found that the aggregated sensitivity of each metabolite $M_{i}$ to all parameters also decreases in the same order (**Figure 2B**). Thus, our results suggest that post-transcriptional regulation of a small number of enzymes is sufficient to maintain homeostasis of IPP/DMAPP biosynthesis in each mutant line.

### 3.6 Investigating average behavior for each mutant type

While having a line-specific, data-driven, model is a more accurate way of describing and predicting the behavior of each mutant line, these are less than helpful in predicting how a new mutant line of any of the three types will behave dynamically.

To create general, type-specific models that are more useful for predicting the dynamic behavior and characteristics of generic new mutant lines, we created a median experimental line for each mutant type. To do so we use the median gene expression activities for each gene in all lines of a given mutant type. Then, we follow the procedure described in methods to generate three new models, one per mutant type. Their steady state concentrations, stability, and sensitivity analysis in **Supplementary Table S10**; **Figure 2**; **Supplementary Data S1**. **Figure 2** also shows that these lines have sensitivity profiles that are similar to those of the individual mutant lines of the same type. Moreover, the models for the median lines of each mutant type have homeostatic behavior that is robust to mutations in enzyme parameters, which is a hallmark of a plausible model.

### 3.7 Variations in whole plant hormone levels partially explain variations in the biosynthesis of IPP/DMAPP in seeds

We also wanted to investigate whether early plant hormone levels in the plant might be a proxy for subsequent changes in the biosynthesis of IPP/DMAPP in seeds. To do so, we calculated how changing hormone levels could explain changes in gene expression and metabolite levels, as described in the methods section 2.11 and in **Supplementary Appendix S1**. We are not assuming that the endogenous MEP, MVA, and exogenous MVA Pathways have an influence in hormone production, only measuring if there is a phenomenological correlation between variations in hormone levels and metabolites or levels of gene expression.

iP is a proxy for changes in enzyme activity in all three mutant types. For all mutant types, iP correlates to changes in the activity of the early MEP pathway steps. In addition, for type I mutants, iP also correlates to changes in the activity of MVA pathway early steps. ABA is also a proxy influence in the early steps of the MEP pathway for all mutant types. Other hormones have a mutant specific effect. **Table S7** summarizes the results for all hormones and presents the hormone dependency equations for each mutant type.

To validate the resulting multilevel models, we investigated if they could reproduce the correlation between experimental hormone levels and model metabolites in the following way.

For each mutant type and metabolite whose concentration is significantly affected by a given hormone, we took the median model described in Section 3.6 and calculated the concentration of the various metabolites as a function of hormone levels. Then, we calculated the correlation in the simulation plot and compared that correlation to the one computed when we plot concentration of the same metabolite *vs* experimental hormone levels. We summarize the results of this analysis in **Table 3**. We find that the models maintain 48 out of 53 expected correlations between hormone levels and metabolite concentrations. This is consistent with the extended models being plausible multilevel descriptions of IPP/DMAPP biosynthesis in the three mutant types.

**Table 3:** Qualitative assessment of expected correlations between metabolites and hormones.

	Number of correlations assessed	Number of correlations matching expected effect	Ambiguous effect or none observed
**Type I**	7	4	3
**Type II**	19	19	0
**Type III**	27	24	2

### 3.8 IPP/DMAPP production increases in mutants with a complete exogenous MVA pathway in the plastid

**Figure 3:**
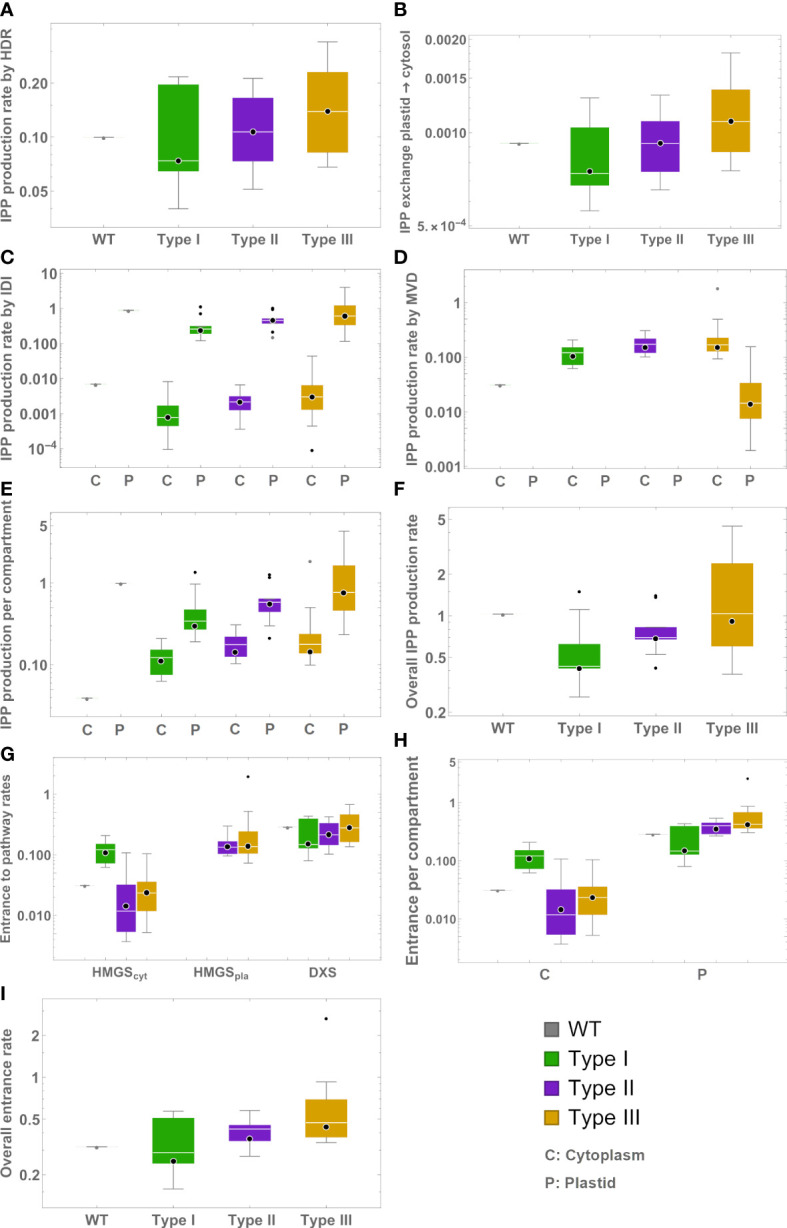
Box plots representing model predictions for exchange and production rates of IPP from different sources. Green – type I mutants. Mauve – type II mutants. Gold – type III mutants. C – Production in cytosol. P – Production in plastid. **(A)** Production by HDR. **(B)** Export to the cytosol. **(C)** Production by IDI **(D)** Production by MVD. **(E)** Total production in each compartment. **(F)** Total production. **(G)** Entry flux to the MVA, MEP, and exogenous MVA pathways. **(H)** Entry flux into IPP/DMAPP production per compartment. **(I)** Overall flux directed for IPP/DMAPP production.

We estimated how the production of IPP/DMAPP changes across mutant lines and types, with the help of the models. **Figure 3** summarizes the results. Overall, our models suggest that median metabolic flux going into the plastid’s IPP/DMAPP producing pathways increases in the following way: Type III>Type II>Type I. We dissected the production rates of IPP by HDR, IDI and MVD (both endogenous and ectopic, where applicable), and the exchange from plastid to cytosol. We also find that the mean production flux for IPP increases from Type I to Type II to Type III.

### 3.9 Plant phenotype can be correlated to hormone and gene expression levels

We used linear regression to investigate if hormone, gene expression, and metabolite levels can predict macroscopic plant phenotypes, such as plant height, number of leaves, leaf width, leaf length and chlorophyll levels, as described in methods. **Table 4** shows that the subset of phenotype parameters that can be predicted from molecular data is different for different mutant types and provides the best fit models for each phenotype parameter and mutant type. Leaf length and chlorophyll content can be predicted for types I, II, and III. Height can be predicted for types I and III. Leaf width can be predicted for types II and III. The number of leaves can be predicted for mutant types I and II. Variations in the levels of the metabolites, genes and hormones shown in **Table 4** are the best predictors for the variations measured in the macroscopic phenotype. The metabolic levels of DXP, MEcPP, HMG-CoA are useful in predicting phenotypical characters of leaves in all mutant types. DMAPP levels are useful in predicting chlorophyll content in type III mutants. **Table 4** also includes the adjusted R^2^ for each model, which is a measure of the percentage of variation in the phenotype that can be explained by the model. Models explain between 40% and 77% of the phenotypical variability, depending on the specific phenotype being measured and the mutant type.

**Table 4:** Multivariate linear models for the phenotype.

		Adjusted R^2^
**Type I**	***Height*** = 56.028 – 22.688 *MDS* + 1.590 *DXP* ***Leaves*** = *Round* (4.738 + 0.318 *DXP* – 0.245 *MEcPP*) ***Leaf Length*** = 31.045 – 3.135 *HMGS* + 1549.320 *HMBPP* ***Chlorophyl*** = 32.218 + 0.154 ***IAA*** – 1.571 *HMGS*	**0.53** **0.72** **0.69** **0.74**
**Type II**	***Leaves*** = *Round* (3.325 – 0.00312 ***ACC*** + 0.0557 *HMGCoA_cyt_*) ***Leaf Length*** = 41.621 + 6.048 *HMGS* – 116.641 *MEP* ***Leaf Width*** = 0.596 + 0.124 *HMGS* + 0.0464 *MVD*1 ***Chlorophyl*** = 39.29 + 1.620 ***iP*** – 1.446 *HDR*	**0.70** **0.77** **0.61** **0.75**
**Type III**	***Height*** = 76.626 + 25.445 ***GA*** 4 + 0.432 *HMGS* ***Leaf Length*** = 50.826 + 0.197 *WR*1 + 9071.25 *MVPP_cyt_* ***Leaf Width*** = 0.881 + 0.005 *HMGS* + 0.007 *HMGCoA_cyt_* ***Chlorophyl*** = 33.218 + 10.968 *HDR* + 75003 *DMAPP*	**0.60** **0.43** **0.40** **0.46**

All units in (cm), except number of Leaves. Blue variables indicate gene expression levels. Bold variables indicate hormone levels. All other variables represent metabolite levels.

### 3.10 Leveraging the models for phenotype prediction

**Figure 4:**
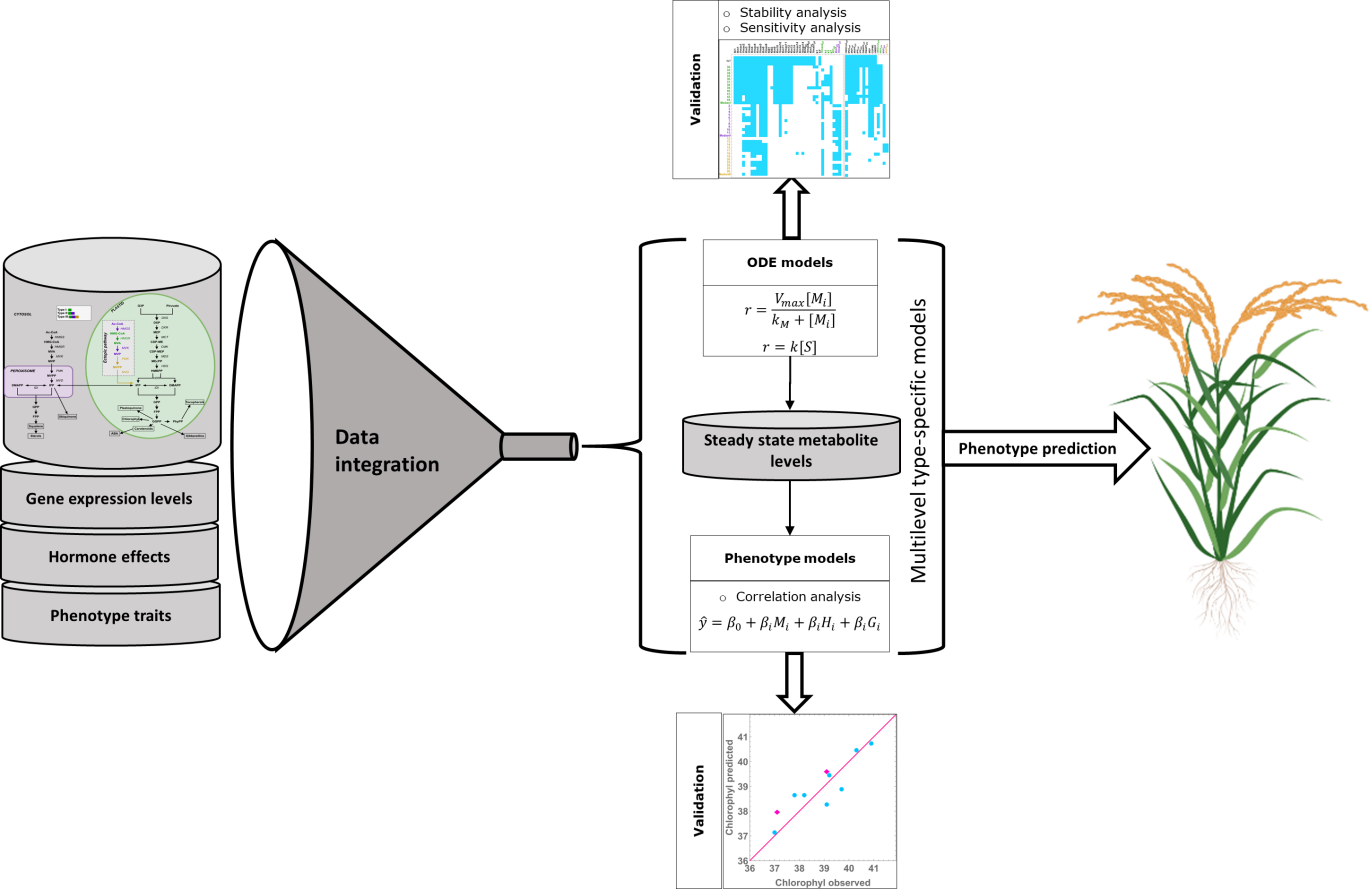
– Multilevel modeling process. We integrate pathway data with gene expression and hormone levels to create line-specific models for isoprenoid biosynthesis. We use the models to calculate metabolic steady state levels, which are then used as input variables, together with hormone levels, to model plant phenotype traits in a type specific manner. We validated the multilevel models and then used them to predict the phenotype of additional rice mutants of each type.

**Figure 5:**
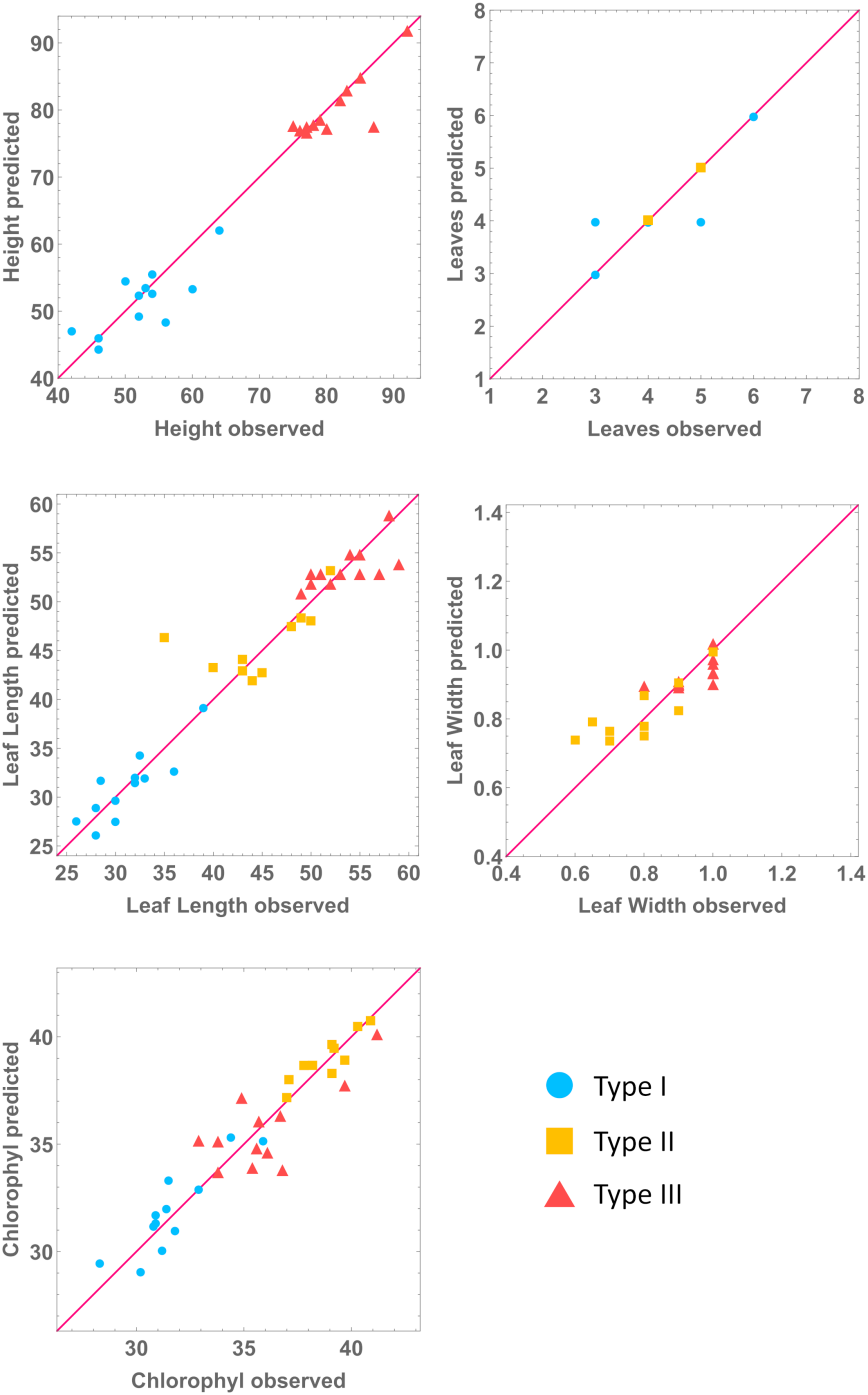
Predicted against observed values of phenotype variables. All units in cm, except chlorophyl (mg/g) and number of leaves. We predict phenotype for each mutant line by combining the molecular line-specific models with the phenotype, type-specific, models. Note: each type is being predicted by its own model.

The results from the previous section allow us to create a multilevel model, connecting metabolite concentrations, and hormone and gene expression levels to the macroscopic plant phenotypes. We summarize the multilevel model building process in **Figure 4**. In **Figure 5** we use the models from **Table 4** and the experimental data for gene expression and hormone levels to calculate what is the expected value for the predicted phenotype, according to the relevant model. Then, we include the experimental determination for the same phenotype. We show that each type-specific model can semi-quantitatively predict macroscopic plant phenotype.

### 3.11 Models are mutant-type specific

**Figure 6:**
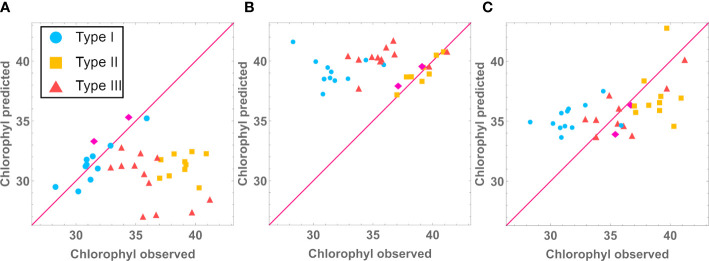
Using Type I **(A)**, Type II **(B)** and Type III **(C)** Chlorophyl models to predict Type I, Type II and Type III chlorophyl levels (mg/g).

We further investigated if the type-specific models were accurate in predicting the phenotype of the other mutant types. We used the median model for each mutant type, feeding it with the experimental determinations for all the lines and measured how accurate the phenotype predictions were for each type. What we observed is that the type-specific models do not properly predict other mutant types. For example, **Figure 6** shows that our type I model can only accurately predict chlorophyll levels for type I lines, failing to do so for mutant types II and III. **Supplementary Table S11** shows that phenotype predictions are only accurate when made with the model for the correct mutant type. This suggests that prediction for new mutant types would require developing a data driven model for that mutant type.

## 4 Discussion

IPP and DMAPP are the precursor monomers to terpenoids, a family of molecules that contains many chemicals with importance in biology, pharmacy, biotechnology, biomedicine and cosmetics, such as squalene. Plants produce those monomers using two biosynthetic pathways: the MVA pathway in the cytosol, and the MEP pathway in the plastid. As such, they are in principle a good substrate for synthetic biology of valuable terpenoid biosynthesis. Plants engineered with exogenous MVA pathway genes are a promising platform for downstream terpenoid production (Andersen et al., 2021; Pérez et al., 2022). For example, when exogenous HMGRS, is expressed in the cytoplasm of tobacco leaves, these leaves appear to produce more cytoplasmic IPP/DMAPP. By also expressing exogenous crtE, crtB, and crtI, the plant uses the excess IPP/DMAPP to become biofortified in carotenoid pigments, such as lycopene (Andersen et al., 2021). Changing the flux going through the MEP plastid pathway has stronger, pleiotropic effects (Pérez et al., 2022). This is likely due to the developmental plant hormones produced from plastid IPP/DMAPP. As such, increasing the production of IPP/DMAPP in the chloroplast while containing the deleterious effects this might have in plant development is more effectively done through expression of an orthogonal MVA pathway in the plastid (Pérez et al., 2022). Still, plants use their IPP and DMAPP to synthesize all their cognate isoprenoids, including developmental hormones and protective molecules. Because of this, engineering plants to divert material from the pathways towards biotechnological purposes has significant pleiotropic effects that are often deleterious (Ye et al., 2000; Ro et al., 2006; Naqvi et al., 2009; Ajikumar et al., 2010; Zorrilla-López et al., 2013; Fuentes et al., 2016; Jiang et al., 2017; Kang et al., 2017; Georgiev et al., 2018; Kotopka et al., 2018; Cravens et al., 2019; Luo et al., 2019; Maeda, 2019; Gülck et al., 2020; Srinivasan and Smolke, 2020; Zhu et al., 2021; Liew et al., 2022; Pérez et al., 2022; Yang et al., 2022; Zhang et al., 2022; Grzech et al., 2023). Consequently, if plants are to be used as a platform for terpenoid biosynthesis, one must engineer IPP/DMAPP biosynthesis in such a way that they are able to properly develop, while producing an excess of monomers that can be used for downstream high value terpenoids production.

This work aims to contribute towards that goal. We generated over thirty independent mutant rice lines that, in addition to the native MVA and MEP pathways, had three alternative versions of an exogenous MVA pathway located to the chloroplast. The method used to create the lines results in a non-targeted integration of the ectopic genes in the genome of the endosperms. This created the potential for different dynamic behavior of pathway metabolites among lines within the same mutant type. We then measured the expression of the genes in the pathways, the hormone levels, and the macroscopic phenotype of the WT and mutant lines. We combined all this data into multiscale, line-specific, mathematical models of the plants that connected all the variables and measurements. We use these models to understand how the alternative versions of the pathways contribute to change the flux going through the IPP/DMAPP metabolic pools.

### 4.1 Modelling limitations

Several modeling efforts focused on analyzing the biosynthetic and signaling dynamics of complex terpenoids in plants (Latowski et al., 2000; Bruggeman et al., 2001; Rios-Estepa et al., 2008; Rios-Estepa et al., 2010; Band et al., 2012; Beguerisse-Diaz et al., 2012; Pokhilko et al., 2013; Pokhilko et al., 2015; Allen and Ptashnyk, 2017; Nazareno and Hernandez, 2017; Dalwadi et al., 2018; Rizza et al., 2021). For example, Band et al. and Rizza et al. use compartmental modeling to study the biosynthesis and diffusion of gibberellins in root tips (Band et al., 2012; Rizza et al., 2021). Terpenoid signaling is often also a target for modeling. For example, Allen & Ptashnyk use models to study signaling interactions between brassinosteroid and gibberellin signaling pathways (Allen and Ptashnyk, 2017), and Nazareno & Hernandez do the same to study signaling interactions between of abscisic acid, ethylene and methyl jasmonate on stomatal closure (Nazareno and Hernandez, 2017). Modeling studies of the biosynthesis and regulation of terpenoid precursors in plants are less common. In fact, we are only aware of two such modeling effort for the MEP pathway in plants (Pokhilko et al., 2015) (Neiburga et al., 2023) and another in *Plasmodium falciparum* (Singh and Ghosh, 2013). The plant MEP model was used to study how circadian rhythms regulate the dynamics of the pathway in plants, while the *P. falciparum* model was used to investigate the regulation of the pathway and to predict the effects of genetic manipulations on the production of isoprenoids with the addition of *in silico* inhibitors. Regarding the MVA pathway, we found no model in plants. Still, this pathway was modeled in bacteria (Weaver et al., 2015, Dalwadi et al., 2018). These MVA models study the dynamics of the pathway in the context of introducing it in bacteria using synthetic biology. Finally, we know of only one other example where both pathways were modeled together in yeast, using Petri net-based dynamic modeling (Baadhe et al., 2012). Taking all this into account, creating models that can be used to study the dynamics and interactions of the MVA and MEP pathways in plants is an important goal, towards which this paper contributes.

Trusting the models normally requires that they are validated by comparison with experimental data that was not used to build them. This was one of the technical limitations in building line-specific models, as we needed most of the available data to estimate the parameter values for each line. As such, the measurements can be used for either model building or model validation, but not both simultaneously. To sidestep this problem, we used three approaches.

First, we used sensitivity and stability analysis as general model quality assessment tools (Savageau, 1975; Kitano, 2007; Voit, 2013) to both evaluate the quality of the line-specific models and identify the parameters that could be used to improve that quality. The WT model is of high quality, being robust to changes in parameters (>92% of parameter sensitivities lower than 0.5) and producing a stable steady state, with metabolite concentrations that are well within the accepted biological ranges (Albe et al., 1990). In contrast, the steady states for the original line specific mutant lines are unstable, leading to unbound accumulation of MEP pathway intermediates. This indicated that the models needed improvement. We hypothesized that the simplest reason for model instability could be consequence of a nonlinear relationship between changes in the expression of pathway genes and changes in enzyme activity. To test if the hypothesis is consistent with our data, we scanned the parameters that represent the enzyme activities that directly produce or use the accumulating metabolites in the model: Vmax9, Vmax12 and Vmax 13. By executing this procedure, we identified the sets of minimal changes to the values for these parameters that generated stable and robust steady states, with metabolic concentrations within known physiological ranges. We remark that other, more complex explanations might also be consistent with the experimental data. Still, Occam’s razor argues that the simplest explanation is the most likely one, in the absence of additional data (Borgqvist and Palmer, 2022; Piasini et al., 2023).

Second, we created “median” models for each of the three types of pathways. To do so, we pooled together all mutant lines of a given type and calculated the median of the pool for each variable. Then, we used that median to create the median model for each mutant type, in the same way we create line specific models. These median models also allowed us to estimate the metabolites for each individual line. Comparing the results with the line specific models shows that these median models have a similar behavior to the models of the individual lines of the same mutant type (**Figure 2**). In addition, when we use the median models to predict the phenotype of the individual lines, the predictions have an error that is similar to that of the individual line models. This suggests that we can use the median model of a given mutant type to study newly created lines for that mutant type.

Third, we used the multilevel models to predict plant phenotype and compare the results with the experimentally determined phenotype, achieving a prediction accuracy of up to 80% (**Supplementary Table S11**). We summarize the process in **Figure 4**. We make different assumptions about the relationship between variables in our modeling. We model the effect of changes in gene expression on the concentrations of pathway intermediates assuming a direct cause-effect relationship between changes in the expression of a gene and changes in the corresponding enzyme activity. We model the effect of enzyme activity on the flux going through the reaction catalyzed by that enzyme using traditional enzyme kinetics. In both cases this assumes a causal relationship between variables. In contrast, we assume that there might be a phenomenological relationship between changes in hormone levels and changes in gene expression and phenotypes and test for that relationship. When we find statistical evidence for that relationship, we include it in our models in different ways. The influence of hormone levels on gene expression is added to the metabolic pathway models through the use of approximation theory, in a way that is mathematically well justified (Salvador, 1996; Sorribas et al., 2007; Alves et al., 2008). The phenomenological influence of hormones, genes, and metabolites on phenotype was accounted for by using statistical linear models. As additional experimental studies become available, the phenomenological parts of the models will need to be adjusted in order to account for the knowledge generated by those experiments. Overall, the quality assessment steps we performed suggest that our models can be used as reasonable semi-quantitative prediction tools to help in better understanding the biology of isoprenoid biosynthesis modification in rice.

### 4.2 Biology of IPP/DMAPP production: from molecular determinants to plant phenotype

Posttranscriptional regulation is important for the proper functioning of the MEP and MVA pathways in plants (Laule et al., 2003; Guevara-García et al., 2005; Sauret-Güeto et al., 2006; Flores-Pérez et al., 2008; Xie et al., 2008; Cordoba et al., 2009; Han et al., 2013). Our modeling and analysis indicate that IPP/DMAPP production is robust to fluctuations in enzyme activity in WT rice (**Table 2**; **Supplementary Tables S8**, **S9**). Further, it suggests that posttranscriptional modulation of enzyme activity is important in stabilizing IPP/DMAPP production in mutant lines (**Figure 2**; **Supplementary Table S2**). In all cases, stabilizing the steady state of a mutant line requires that the activity of a protein is upregulated with respect to the changes in gene expression for that protein (**Supplementary Data S1**). This is fully consistent with the observation that, when compared to the WT *Arabidopsis* plants, changes in the activities of DXS and DXR proteins are bigger than the changes in expression of the corresponding genes (compare panels A and C of **Figure 3** in Flores-Pérez et al. (2008)). Interestingly, DXR is one of the proteins flagged in our models as a potential stabilizing influence for the steady state of the rice mutants (**Supplementary Data S1**).

Our results also suggest that variations in plant hormone levels can predict, to some extent, both plant phenotype (**Table S11**) and the biosynthetic fluxes of IPP/DMAPP in the seeds (**Table S7**). Further, variations in plant gene expression levels in combination with variations in plant hormones can improve phenotype predictions (**Tables 4**, **S11**). However, the more complex the genetic manipulation was, the less accurate the phenotype predictions become. While the median adjusted R^2^ for the predictions is approximately 60% in mutant types I and I, this number goes down to 35% in type III mutants (**Table S11**).

Pérez et al. (2022) also reported that plant development is more similar to that of the WT in mutant type III, followed by mutant type II, and finally I. Our modeling suggests an explanation for this observation. The analysis predicts that the global production of IPP/DMAPP is on average higher in the mutant types that have a more complete version of the exogenous MVA pathway in the plastid (**Figure 3**). The average amount of flux going through the endogenous MEP and MVA pathways in Type III mutants is the most similar to that of the WT, followed by the flux going through the endogenous pathways in Type II mutants. The least similar flux to WT is that of Type I mutants (**Figure 3**). **Figure 3** also shows that the total amount of flux going through the IPP/DMAPP pools in mutant types I and II is similar to that of the WT. The flux going through IPP/DMAPP is larger in Type III mutants than in the WT rice. Taken together, these observations suggest that plants can distinguish between the IPP/DMAPP produced by each of the pathways. Too little flux going through the endogenous MEP pathway compromises the production of developmental hormones leading to plants with developmental defects. In conclusion, we believe that an iterative modeling-experimental process as the one presented here would be an effective way to identify which parts of each pathway are more sensitive to further manipulation, and which parts are more likely to be good targets for modification in order to increase the flux without disrupting the development of the plant.

## Data availability statement

The original contributions presented in the study are included in the article/**Supplementary Material**. Further inquiries can be directed to the corresponding author.

## Author contributions

OB and RA designed the modelling and performed the in silico analysis. LP, PC and TC designed the plasmids, transformed and recovered the plants, and measured phenotypes. LF and PF designed and performed the metabolomics experiments. AA and CA designed and performed the hormone measurements. OB, RA, AL, EV, AM-S and AS analyzed results. OB and RA wrote the paper. All authors contributed to the article and approved the submitted version.
