# Supplementary material for: Changing biosynthesis of terpenoid percursors in rice through synthetic biology
Source: Front Plant Sci. 2023 Jul 3;14:1133299. doi: 10.3389/fpls.2023.1133299 (PMC10350630; doi:10.3389/fpls.2023.1133299)
Supplement: Supplementary file 1 [file DataSheet_1.pdf]

Supplementary Appendix S1 for Changing biosynthesis of terpenoid precursors in rice through synthetic biology, by Basallo et al.

Contents

Contents 1
Chapter 1. Methods 3
1.1. Mathematical Modeling 3
1.2. The endogenous MVA and MEP pathways 3
1.3. The ectopic MVA pathway in plastid 3
1.4. Exchange of MVA and MEP pathway metabolites between the cytoplasm and the plastid 3
1.5. Estimating rate constants, metabolite concentrations and variations in enzyme activity 3
1.6. Assembling the models and solving the differential equations 4
1.7. Stability analysis 4
1.8. Sensitivity analysis 5
1.9. Including hormone influence in the models 5
1.10. Phenotype models 6
1.11. Gene constructs and gene expression 7
1.12. Hormone determinations 8
Chapter 2. Results 9
2.1. Gene expression measurements 9
2.2. Hormone determinations 10
2.3. Phenotype measurements 11
2.4. Sensitivity Analysis of the WT line 12
2.5. Stabilization of the mutant models 12
Chapter 3. Discussion 12
Chapter 4. References 15

Chapter 1. Methods

1.1. Mathematical Modeling

We used ordinary differential equation systems to model the biosynthesis of IPP/DMAPP. The mathematical formalism used to describe the flux dynamics is the saturating cooperative formalism (Alves et al., 2008; Sorribas et al., 2007). This formalism allows us to approximate the kinetics of any given reaction using a rational expression, where parameters have physical interpretations that are common in enzyme kinetics. Vmax parameters represent the apparent rate constants of the reactions. Kms represent the apparent Michaelis-Menten constants for the substrate(s). K_is represent the apparent inhibition constants of inhibitors. While no activators were considered in our model, these can also be included using this formalism.

1.2. The endogenous MVA and MEP pathways

To model the wildtype (i.e., the endogenous MVA and MEP pathways), we used the reactions shown in Figure 1 and modelled the kinetics of each process using the rate expressions in Table S1 and Table S2, as well as the exchange fluxes of IPP and DMAPP between cytoplasm and plastid (Table S4). We assume that the cell maintains homeostasis of Acetyl-CoA and Acetoacetyl-CoA.

1.3. The ectopic MVA pathway in plastid

The difference between the WT and mutant rice lines is that the mutant rice lines have genes that code for plastid-localized enzyme versions of the MVA pathway.

To model type I mutants, we added the reaction that transforms HMG-CoA_{pl} into MVA_{pl} to the plastid (Table S3), as well as the cytoplasm-plastid exchange reactions for these two metabolites (Table S4). In Figure 1, we sequentially added ectopic MVA pathway from type I (only the green), to type II (adding green and purple), and to type III (whole ectopic pathway)

1.4. Exchange of MVA and MEP pathway metabolites between the cytoplasm and the plastid

Under physiological conditions, IPP and DMAPP flow from the plastid into the cytosol in a mostly unidirectional way (Bick & Lange, 2003). We implemented this observation by assuming that metabolites flow from the plastid to the cytosol at ten times the rate of the import reaction from the cytosol. Bick and Lange (Bick & Lange, 2003) also reported that other pathway intermediates were not actively transported between the two compartments. Nevertheless, early intermediates of the MVA pathway can be found in the plastid space (Schneider et al., 1977). As such, we assumed that HMG-CoA, MVA, MVP and MVPP enter and leave the plastid, albeit at slower rates. Table S4 summarizes all reactions of material interchanged between plastid and cytosol.

1.5. Estimating rate constants, metabolite concentrations and variations in enzyme activity

Table S5 presents the rate constants for each reaction in the models. Table S6 presents the concentrations for the independent variables of the model.

We extensively searched the literature for quantitative and qualitative information about the correlation between changes in gene expression and enzyme activities in the MEP and MVA pathways. As we found no such information, we modeled variations in the enzyme activities of the mutant lines as described in Comas et al. (2016): changes in gene expression with respect to the WT are assumed to be proportional to changes in protein activity. This is the simplest

possible assumption about the relationship between changes in gene expression and changes in enzyme activity.

We explicitly consider the enzymes that catalyze each reaction in the rate expressions. As $V_{max} \approx k_{cat}Enzyme$, the model for the WT sets the enzyme activity to be 1 (the basal state). As we model mutant lines we assume that changes in gene expression are proportional to changes in enzyme activity and make $Enzyme = Enzyme_{WT}\frac{Gene\ expression\ in\ mutant\ line}{Gene\ expression\ in\ WT}$.

The values for the ectopic pathway enzymes are set to zero if the gene is absent. This ensures the reaction does no take place in the model. When they are present, $Enzyme = Gene\ expression\ in\ mutant\frac{line}{Gene}expression\ in\ the\ WT$.

1.6. Assembling the models and solving the differential equations

Each metabolite has its own differential equation in the model. The kinetic rate function, f_j, for each process that produces a metabolite M appears as a positive term in the differential equation that determines the dynamic behavior of that metabolite. Similarly, the kinetic rate function, f_k, for each process that consumes a metabolite appears as a negative term in the differential equation that determines the dynamic behavior of that metabolite:

$$\frac{dM}{dt} = \sum f_j - \sum f_k \tag{1}$$

For each line of rice, we assemble a system of ordinary differential equations (ODEs) that describes the dynamic behavior of all metabolites in the system. To solve the ODEs, we assume that the metabolic concentrations are in rapid equilibrium with respect to the changes in gene expression (Comas et al. 2016). Because of that, we solve the systems of differential equations for the steady state of the pathways.

1.7. Stability analysis

Biological steady states should be stable (Savageau, 1975; Voit, 2013). Stability measures the capacity of a system to remain or return to the steady-state. Lack of stability indicates that the model is missing information (Savageau, 1975; Voit, 2013).

As such, and to validate or correct our modeling assumptions, we performed a stability analysis of the models for each rice line.

An efficient way to assess stability is by calculating the eigenvalues of the Jacobian matrix of the ODE system, which are complex numbers (Voit, 2013). If all real parts of all eigenvalues are negative, the system is stable. Otherwise, the system is unstable. The Jacobian matrix is constructed by taking the partial derivatives of the right-hand side of the ODEs (f_i) with respect to each state variable (x_j), as shown in Eq. 2.

$$J = D_x f = f_x = \frac{\partial f_i}{\partial x_j} = \begin{pmatrix} \frac{\partial f_1}{\partial x_1} & \frac{\partial f_1}{\partial x_2} & \cdots & \frac{\partial f_1}{\partial x_n} \\ \frac{\partial f_2}{\partial x_1} & \frac{\partial f_2}{\partial x_2} & \cdots & \frac{\partial f_2}{\partial x_n} \\ \vdots & \vdots & \ddots & \vdots \\ \frac{\partial f_n}{\partial x_1} & \frac{\partial f_n}{\partial x_2} & \cdots & \frac{\partial f_n}{\partial x_n} \end{pmatrix} \quad (2)$$

1.8. Sensitivity analysis

In addition to stable, biological steady states should be robust, and have low sensitivity to parameter changes (Savageau, 1975; Voit, 2013). Sensitivity measures how much a dependent variable or output changes when a parameter is altered (Comas et al., 2016). Parameters with high sensitivities indicate regions of the network that are incomplete or inaccurate.

As such, we performed a sensitivity analysis to investigate the robustness of the models and the places in the network where our assumptions may have been too simplifying. This allowed us to identify which steps of the pathway could have additional regulation that we were ignoring.

We calculated logarithmic, or relative, steady-state parameter sensitivities, which measure the relative change in a system variable (X) that is caused by a relative change in a parameter (p)" (Voit, 1991):

$$\bar{S}(X,p) = \frac{\partial X/X}{\partial p/p} = \frac{\partial \log X}{\partial \log p} \quad (3)$$

1.9. Including hormone influence in the models

Hormones were included in the models using a three-stage process.

First, and for each pair of metabolite-hormone, we adjusted a linear model that assumes the metabolite is a function of the hormone. We calculate the adjusted R^2 of the models and filter out all metabolite-hormone pairs with adjusted R^2 below 0.20.

Second, for the remaining metabolite-hormone pairs after filtering, we investigated how best to account for hormone influence in the ordinary differential equations. We tested two alternative mathematical formalisms.

On the one hand, we considered the power-law formalism. This is a simple non-linear formalism that can be used to approximate unknown flux functions and has strong theoretical support (Alves et al., 2008; Sorribas et al., 2007). In this situation

$$M_i = \alpha H_j^{g_{ij}}, \quad (4)$$

Where M_i is the concentration of metabolite *i* in the model, H_j is the level of hormone *j* at twelve weeks, and α and g are constants. This formalism can be linearized as

$$\log M_i = c + g_{ij} \log H_j \quad (5)$$

where $c = \log \alpha$.

On the other hand, we considered the saturating cooperative formalism, which is a more detailed approximation in situations where saturation needs to be described (Alves et al., 2008; Sorribas et al., 2007). Using this formalism, we can write

$$M_i = \alpha \left(\frac{H_j}{K + H_j} \right)^{g_{ij}}, \tag{6}$$

where K is the concentration of hormone that produces half the effect. We linearized it for linear model fitting as

$$\frac{1}{\sqrt[g_{ij}]{M_i}} = \frac{1}{d} + K \frac{1}{d\, H_j}, \tag{7}$$

where $d = \sqrt[g_{ij}]{\alpha}$.

To select which formalism to use when including hormone influence in each line we used a criterion that considers the adjusted R^2 and the value for g_{ij}. A combination of low adjusted R^2 and high $|g_{ij}|$ suggests a potentially strong influence of the hormone levels on metabolite concentrations (high d $|g_{ij}|$) over a small range of hormone levels (low adjusted R^2). In this situation, we assumed a saturation effect and used the saturating cooperative formalism to model hormone influence on metabolite production and consumption. Otherwise, we used the power law formalism, as it uses a smaller number of parameters and minimizes the possibility of overfitting the model to the data. The threshold for selecting the one or the other formalism was set at 0.5 for the ratio $|R^2_{adj}/g|$. If $|R^2_{adj}/g| > 0.5$ we use the power law formalism. Otherwise, we use the saturation cooperative formalism. The resulting formalisms are summarized in Table S7.

The final step was selecting the flux to which the hormone influence should be included in. To decide this, we calculated the correlation between gene expression levels and hormones using a standard linear model

$$G_i = a + bH_j, \tag{8}$$

where G_i is the gene expression levels at 12 weeks. If a hormone was correlated to a metabolite, and at the same time was correlated to expression levels of a gene involved in the production or consumption of said metabolite, we multiplied the hormone dependency function by the flux function for the process catalyzed by the gene. If no strong and significant correlation was found, we multiplied the hormone dependency term by the overall production (consumption) flux of the metabolite if the correlation between hormone and metabolite was positive (negative).

We note that, when hormones were below the experimental detection threshold, we revert the kinetic expression presented in Table using a piece-wise approximation to solve the differential equations.

1.10. Phenotype models

We took an algebraic approach to model different phenotype variables as a function of hormones, genes and intermediate metabolites of terpenoid biosynthesis. The phenotypic characteristics to be modeled were the plants' *Height*, number of *Leaves*, *Leaf Length*, *Leaf*

Width, and *Chlorophyl* levels. Data is separated according to mutant type, so the analysis and model building is performed three times, one for each mutant type.

First, we built linear models with one predictor variable and selected the predictor variables that had a significant ($\alpha = 0.05$) effect on the phenotype and whose model had an Adjusted R^2 greater than 0.2. With the remaining phenotype variables and predictors, we built every possible multivariate model with all subsets of predictors. So, for a given subset of predictors $\{x_1, x_2, \dots, x_n\}$, the model would be:

$$\hat{y} = \beta_0 + \beta_1 x_1 + \beta_2 x_2 + \cdots + \beta_n x_n \tag{9}$$

To avoid overfitting, we compared the models using AICc (AIC corrected for small sample sizes) and BIC estimators. In addition to AICc and BIC, the p-values of the multiple predictors' effects and the adjusted R^2 were considered when the best multivariate model was selected.

1.11. Gene constructs and gene expression

Table A summarizes the transgenes used to create a plastidic MVA pathway.

Table A: Gene constructs

Transgene	Original organism	Promoter/terminator
BjHMGS	*Brassica juncea*	*Hordeum vulgare* D-hordein promoter and *Agrobacterium tumefaciens* nopaline synthase (nos) terminator
tHMGR	*Arabidopsis thaliana*	Triticum aestivum low-molecular-weight glutenin promoter and rice ADPGPP terminator
CrMK	*Catharanthus roseu*	*H. vulgare* D-hordein promoter and *A. tumefaciens* nos terminator
CrPMK	*Catharanthus roseu*	*Zea mays* γ-zein promoter (GZ63) and *A. tumefaciens* nos terminator
CrMVD	*Catharanthus roseu*	*Orizae sativa* prolamin promoter (RP5) and *A. tumefaciens* nos terminator

We assembled BjHMGS, tHMGR, CrMK, CrPMK, CrMVD into two separate expression vectors. We inserted a transit peptide at the N-terminal region of the enzymes in order to direct them to the plastid. GenScript (Piscataway, NJ, USA) optimized the sequences of the transgenes for expression in rice and pre-assembled them onto two vectors. Vector one contained the three initial genes of the MVA pathway (BjHMGS:tHMGR:CrMK). Vector two contained the final two genes of the MVA pathway (CrPMK:CrMVD). An additional plasmid containing the hygromycin phosphotransferase (hpt) selectable marker gene (Christou et al. 1991) under the control of the constitutive cauliflower mosaic virus 35S promoter and the nos terminator to select the transgenic rice plants.

We used the RNeasy Plant Mini Kit (Qiagen, Hilden, Germany) to isolate total seed RNA, which we quantified using a Nanodrop 1000 spectrophotometer (Thermo Fisher Scientific). We used 2 µg of total RNA as a template for first strand cDNA synthesis with Quantitech reverse transcriptase (Qiagen) in a 20-µL reaction volume. We used a CFX96 system (Bio-Rad, Hercules, CA, USA) for Real-time qRT-PCR, using 20-µL mixtures containing 5 ng cDNA, 1 × iQ SYBR Green Supermix and 0.5 µM of the forward and reverse primers designed for the transgenes BjHMGS,

tHMGR, CrMK, CrPMK, CrMVD, and the endogenous MVA and MEP pathway genes OsHMGS, OsHMGR, OsMK, OsPMK, OsMVD, OsDXS, OsDXR, OsMCT, OsCMK, OsMDS, OsHDS, OsHDR and OsIPPI . We used serial dilutions of cDNA (80–0.0256 ng) to generate standard curves for each gene. We performed triplicate PCR using 96-well optical reaction plates. We plotted the ΔCt values of different primer combinations of serial dilutions against the log of starting template concentrations using CFX96 software to calculate amplification efficiencies. We used the rice housekeeping OsActin1 (ABF98567.1) as an internal control.

1.12. Hormone determinations

We built calibration curves for each phytohormone (1, 10, 50 and 100 µg L−1) and corrected for 10 µg L−1 deuterated internal standards. These curves were used to quantify the hormones in transgenic rice lines.

We homogenized 0.1 g of fresh leafs in liquid nitrogen. Then, we immersed the homogenate in 1 mL 80:20 (v/v) methanol/water at –20 °C, separating the solids through centrifugation (20,000 × g, 15 min, 4 °C) and re-extracted for 30 min at 4 °C as above. We separated the pooled supernatants using Sep-Pak C18 Plus cartridges (Waters, Milford, MA, USA), thus removing interfering lipids and pigments. Finally, we evaporated the mix under vacuum (40 °C) to near dryness. We then dissolved the residue in an ultrasonic bath containing 0.5 mL 20:80 (v/v) methanol/water. We passed the dissolved samples through 0.22-µm Millex nylon filters (Millipore, Bedford, MA, USA), and injected 10 µL of the filtered extract in an Accela Series ultra-high-performance liquid chromatography (UHPLC) system coupled to an Exactive mass spectrometer (Thermo Fisher Scientific, Waltham, MA, USA) via a heated electrospray ionization (HESI) interface. We used Xcalibur v2.2 (Thermo Fisher Scientific) to obtain the mass spectra. We achieved 92–95% target compound recovery.

Chapter 2. Results

2.1. Gene expression measurements

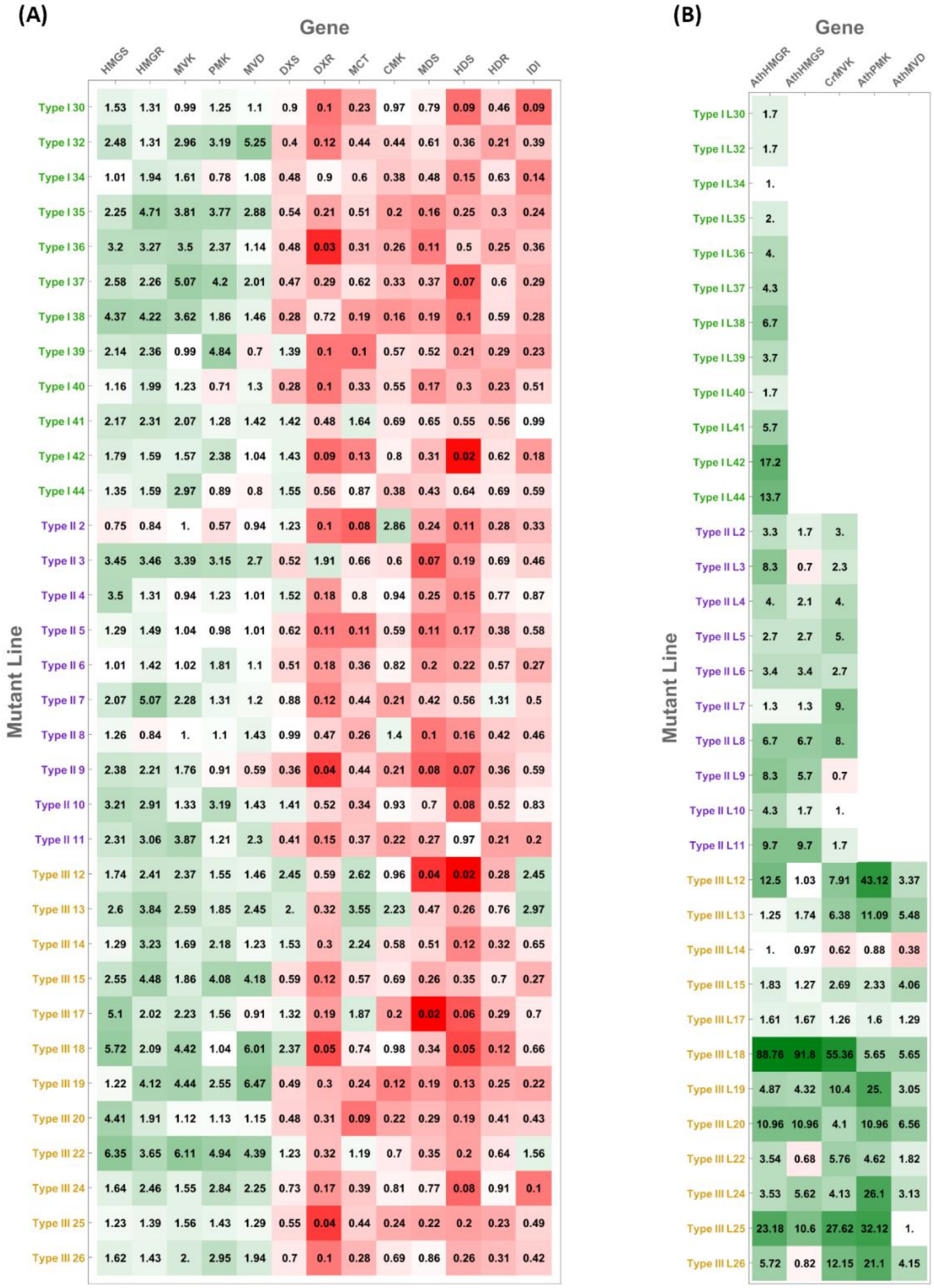

Figure A. Gene expression levels. (A): Endogenous genes, normalized with respect to the gene expression in WT rice. We normalized WT gene expression with respect to actin levels (B): Exogenous genes, normalized with respect to actin levels.

2.2. Hormone determinations

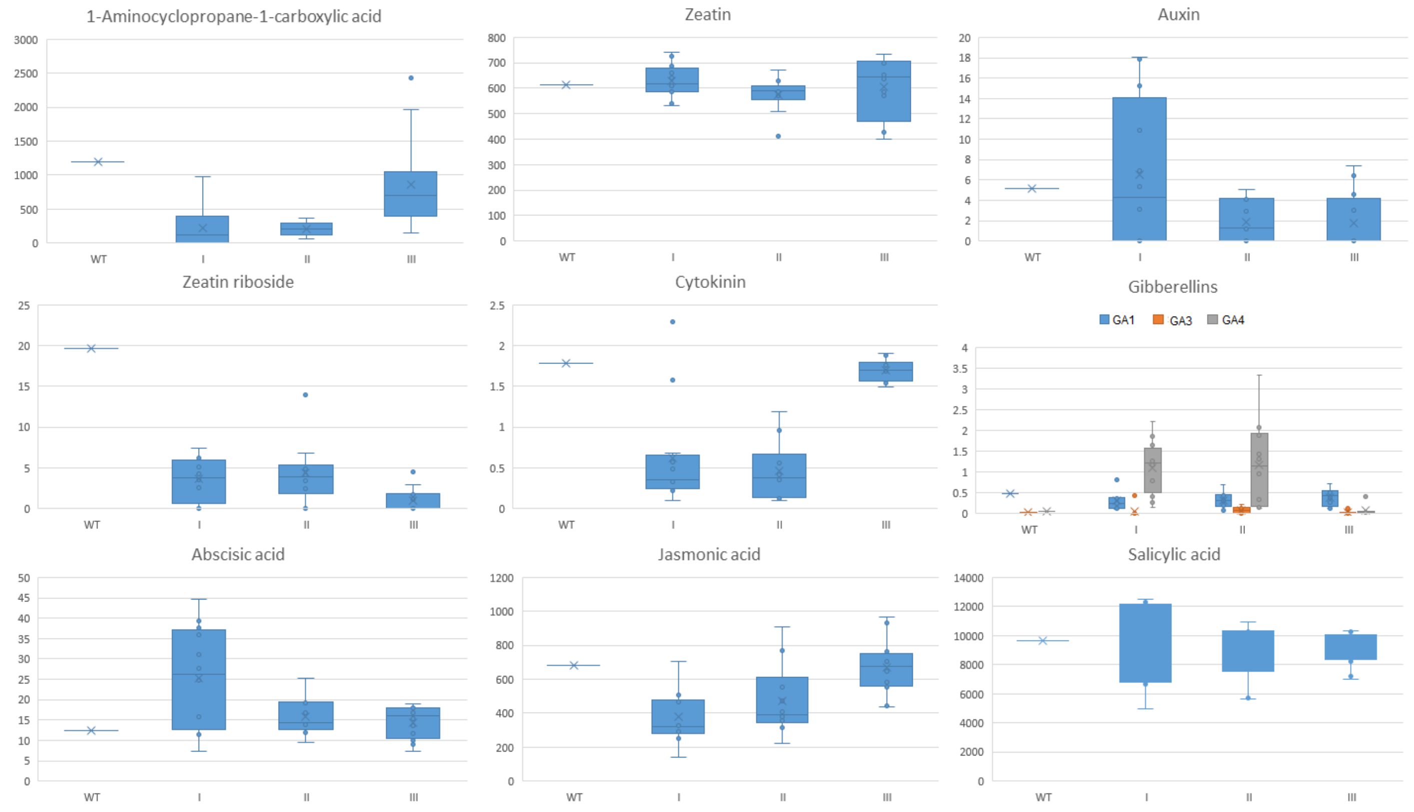

Figure B – Experimental measurements for plant hormones in the four types of rice lines. See section 1.12 for technical details.

2.3. Phenotype measurements

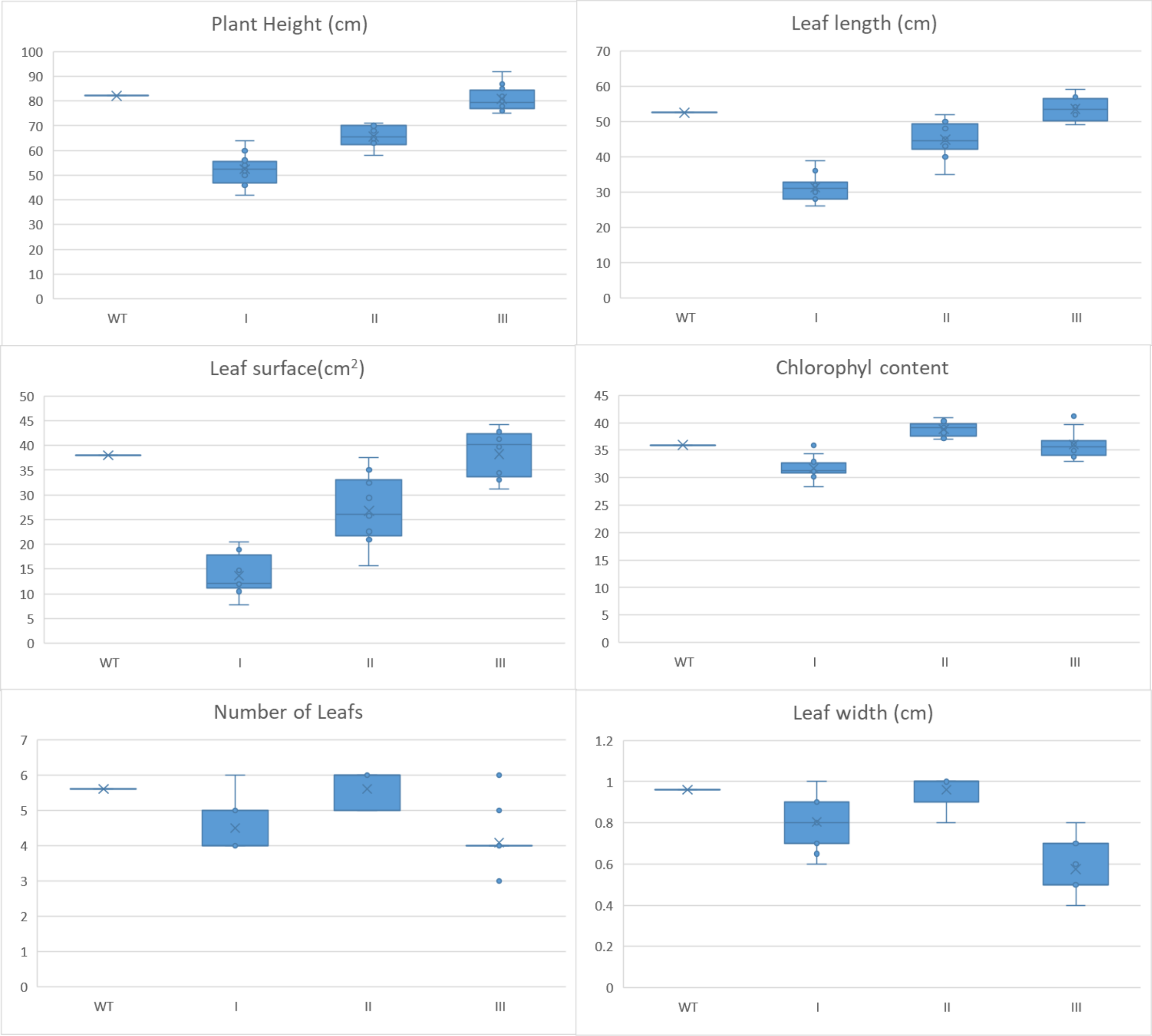

Figure C - Phenotype measurements. Phenotypes were determined as described in the main text.

2.4. Sensitivity Analysis of the WT line

Sensitivity analysis identifies the parameters to which the various variables of the model are most sensitive, as described in (Alves et al., 2008; Sorribas et al., 2007). A high sensitivity of a variable to a parameter indicates that small changes in that parameter might lead to big changes in the variable.

We performed a logarithmic sensitivity analysis of the dependent concentrations of the WT model with respect to each parameter of the model. Table S9 shows the concentration sensitivities. We only present sensitivities with an absolute value larger than 0.5.

2.5. Stabilization of the mutant models

Biological steady states should be stable and robust, having low sensitivity to parameter changes (Savageau, 1975; Voit, 2013). When implementing the modified models for each mutant line as described in sections 2.3 to 2.6, we found that the steady states were unstable for some of those lines, with a few intermediate metabolites accumulating indefinitely. Unstable steady states indicate that the models are not adequate representations of the biological situation (Savageau, 1971, 1976).

One possibility for explaining this instability is the existence of a non-proportional relationship between changes in gene expression and changes in enzyme activity (see section 2.5). To test this hypothesis, we investigated if the steady state of a line would stabilize if changes in enzyme activities and changes in gene expression were not proportional.

Using a minimal intervention policy, we identified the metabolites that accumulated in each line, which were DXP, CDP-MEP, MEcPP, or combinations of them. Reactions r9, r12, and r13 of Table S2 either produce or consume these metabolites. Consequently, we scanned the values for the Vmax parameters of reactions Vmax9, Vmax12 and Vmax13 in order to identify the minimum change in those parameters that would stabilize the steady state of each mutant line.

To find the values of Vmax that made the models stable we scanned one-dimensional, two-dimensional and three-dimensional spaces, and we checked for steady state stability with each set of parameter values. The sets of Vmax that made the models stable were stored in a candidate sets list and we chose the best as the one with minimum Euclidian distance to the original set of values of that line. Figure S1 shows that, overall, the more complex the model is, the bigger must the change in parameter values be to stabilize the model.

Chapter 3. Discussion

The terpenoid family holds many chemicals with importance in pharmacy, biotechnology, biomedicine and cosmetics. IPP and DMAPP production and polymerization is key to their biosynthesis. Plants produce these monomers with two pathways: MVA pathway (cytosol) and MEP pathway (plastid). A part of the flux is used for the biosynthesis of developmental hormones, making redirection of the flux detrimental to the plant. Developing plants that produce IPP/DMAPP in high amounts is a requirement for their use as a platform to produce high value terpenoids, and an important goal in biotechnology. We participated in the creation of mutant rice lines that express the totality or parts of the MVA pathway in the plastid. Data was collected on the macroscopic and molecular phenotypes, gene expression, terpenoid

compounds and hormone levels of the different mutant lines. Intermediates of the pathways could not be measured with the current technology. Integrating the different data and understanding the changes brought by the ectopic pathway is complex. To generate understanding of this, we developed line specific mathematical models using the available data.

We used ODEs to model terpenoid biosynthesis. We described the reactions in the pathways with cooperative formalisms, and power laws for diffusion rates, with a couple of exceptions where data was not available. For the different mutants, the ectopic pathway reactions were included as corresponded to the type of mutant and line specific Vmax were given by directly correlating gene expression data to enzyme activity, so that the new Vmax were the WT model Vmax multiplied by the factor of increase/decrease of gene expression with respect to the WT line. We integrated the hormone effects by searching correlation between them and the metabolites and representing the effect as either a power law or a saturation formalism depending on the strength and significance of the correlation.

The WT model, or basal, model passed all generic quality controls for model behavior. The steady state of the WT model was stable, as proved by the eigenvalues. Out of 700 sensitivities variable-to-parameter calculated, 646 were lower than 0.1. An example of high sensitivity is IPP_{pla} when Km6 is varied. From the sensitivities of eigenvalues to parameters, 677 out of 700 were below 0.1. High sensitivities help identify places where the model can be improved, because such places act as weak links of a chain towards stability and robustness of the steady state values. The concentrations given by its steady state are also within ranges that make biological sense. Even if the current technology does not allow to validate this experimentally, due to these intermediates having a short residence time, the models allow us to see differences on the steady states based on the gene expression levels. The changes in metabolites propagate as expected when one flux is increased or reduced, with the model being more susceptible to instability in the MEP pathway, due to feedback regulation only existing in the first step of the pathway. The models tell us that, due to the differences in gene expression and the ectopic pathway, the IPP and DMAPP levels should decrease in the mutant lines.

We scanned Vmax9, Vmax12 and Vmax 13 to make the mutant models stable. For each line, we detected which of those three metabolites accumulated, and the corresponding Vmax was tweaked. This “simplest solution” approach would not work alone, because increasing upstream rates was causing the problem to be displaced downstream (e.g., if only DXP was accumulating and we increased its depletion rate, then CDP-MEP would accumulate instead). Therefore, the Vmax downstream were scanned as well. We tried to stabilize by decreasing Km9, Km12 and Km13 instead, but the impact was not enough to avoid metabolite accumulation, so we discarded this approach. Our analysis of sensitivity of eigenvalues to parameters would make us expect that at least changing Vmax12 and Vmax13 would affect the stability of the model. However, the eigenvalues that become destabilized do not correspond with DXP, CDP-MEP, MEcPP. Such metabolite specific correlation could be hidden in multivariate sensitivity, as stabilizing often required changing more than one of those parameters.

Our models assume that changing gene expression directly correlates to changing enzyme activities. That, together with the fact that Vmax9, Vmax12 and Vmax 13 required adjustment, could imply that regulation not described in literature is required at those steps of the pathway. Said regulation would cause the decoupling between gene expression and protein synthesis or be post-translational regulation of protein activity.

The method used in the transformation of the lines results in a non-targeted integration of the ectopic genes in the genome of the endosperms. This could explain different behaviors between lines within the same mutant type. While some lines are unstable and others are not within the

same type, once stabilized, the difference in metabolite levels among lines is usually not greater than one order of magnitude. The model is restricted to the MVA and MEP pathways, and squalene, sterols and Vitamin E could not be integrated as we were missing transcriptomic data on the enzymes responsible for the creation and consumption of said metabolites.

The lack of data for testing makes impossible for now to validate the model quantitatively. Metabolite data is obtained in-silico from the models without the hormone effects, so we could not use it for validation of the final models. However, metabolite-hormone correlation could be carried over from the steady state simulations in case where gene expression-hormone correlation exists. That is, the models without the hormones are still implicitly correlating metabolite levels to gene expression (even though at a multivariate scale), so if expression-hormone correlation is found, hormone to metabolite effects could be inferred.

Additionally, non-expected tendencies with other metabolites are observed when adding the hormone effects. Different hypothesis could be attributed to this observation. First, these correlations were filtered out with the adjusted R^2. This could be detected with more data points, or by trying different thresholds for filtering candidate hormone effects. Second, these correlations propagate from upstream or downstream effects. The fact that one correlation is detected but not others could imply additional regulation in the pathway. Our model would not reflect those regulations and therefore effects would propagate to other metabolites proportionately. Testing different models that have been modified to include such regulation could be a way to detect this. Alternatively, removing one source of variation of our model and focusing on the factor of interest could help. Our models have two sources of variation: one is the variables and rates considered in the specific mutant-type model (the “shape” of the model itself), and the other is the different Vmax values given to each line, which are directly correlated to gene expression. For example, there is no reason to not include the plastid versions of MVA pathway intermediates in the WT model, other than Ockham’s Razor, as we consider to be exchange between cytosol and plastid.

As for the phenotype models, the predicted versus observed plots give us an idea of the performance of the selected linear models, thought we need to mind that part of the data (hormones and gene expression) is the same that was used to build the models. In other words, we lack a traditional test set. Predicted values are in the range of the real values, and Type I models perform well. The performance seems progressively worse in Type II and then Type III models. This drop in performance could be explained as the increasing complexity of the ODE models adding noise to the metabolite *in silico* data, but some of the low performing linear models do not include metabolite predictors at all, ruling out that possibility. Thus, the building of the models itself is faulty, as confirmed by the lower significance of the predictors’ coefficients in those models. The tendency of the low performing models is to overestimate lower values of the independent variable and underestimate higher values.

Chapter 4. References

Albe, K. R., Butler, M. H., & Wr1ghtt, B. E. (1990). Cellular Concentrations of Enzymes and Their Substrates. In *J. theor. Biol* (Vol. 143).

Alves, R., Vilaprinyo, E., Hernádez-Bermejo, B., & Sorribas, A. (2008). Mathematical formalisms based on approximated kinetic representations for modeling genetic and metabolic pathways. *Biotechnology & Genetic Engineering Reviews*, *25*, 1–40. http://www.ncbi.nlm.nih.gov/pubmed/21412348

Bach, T. J., & Benveniste, P. (1997). Cloning of cDNAs or genes encoding enzymes of sterol biosynthesis from plants and other eukaryotes: Heterologous expression and complementation analysis of mutations for functional characterization. *Progress in Lipid Research*, *36*(2–3), 197–226. https://doi.org/10.1016/S0163-7827(97)00009-X

Benveniste, P. (1986). Sterol Biosynthesis. *Annual Review of Plant Physiology*, *37*(1), 275–308. https://doi.org/10.1146/annurev.pp.37.060186.001423

Bick, J. A., & Lange, B. M. (2003). Metabolic cross talk between cytosolic and plastidial pathways of isoprenoid biosynthesis: unidirectional transport of intermediates across the chloroplast envelope membrane. *Archives of Biochemistry and Biophysics*, *415*(2), 146–154. https://doi.org/10.1016/S0003-9861(03)00233-9

Comas, J., Benfeitas, R., Vilaprinyo, E., Sorribas, A., Solsona, F., Farré, G., Berman, J., Zorrilla, U., Capell, T., Sandmann, G., Zhu, C., Christou, P., & Alves, R. (2016). Identification of line-specific strategies for improving carotenoid production in synthetic maize through data-driven mathematical modeling. *The Plant Journal*, *87*(5), 455–471. https://doi.org/10.1111/TPJ.13210

Dharmapuri, S., Rosati, C., Pallara, P., Aquilani, R., Bouvier, F., Camara, B., & Giuliano, G. (2002). Metabolic engineering of xanthophyll content in tomato fruits. *FEBS Letters*, *519*(1–3), 30–34. https://doi.org/10.1016/S0014-5793(02)02699-6

Eisenreich, W., Rohdich, F., & Bacher, A. (2001). Deoxyxylulose phosphate pathway to terpenoids. *Trends in Plant Science*, *6*(2), 78–84. https://doi.org/10.1016/S1360-1385(00)01812-4

Enfissi, E. M. A., Fraser, P. D., Lois, L.-M., Boronat, A., Schuch, W., & Bramley, P. M. (2004). Metabolic engineering of the mevalonate and non-mevalonate isopentenyl diphosphate-forming pathways for the production of health-promoting isoprenoids in tomato. *Plant Biotechnology Journal*, *3*(1), 17–27. https://doi.org/10.1111/j.1467-7652.2004.00091.x

Fraser, P. D., Romer, S., Shipton, C. A., Mills, P. B., Kiano, J. W., Misawa, N., Drake, R. G., Schuch, W., & Bramley, P. M. (2002). Evaluation of transgenic tomato plants expressing an additional phytoene synthase in a fruit-specific manner. *Proceedings of the National Academy of Sciences of the United States of America*, *99*(2), 1092–1097. https://doi.org/10.1073/pnas.241374598

Gylling, H., & Miettinen, T. (1999). Phytosterols, analytical and nutritional aspects. In R. Lasztity, W. Pfannhauser, L. Simon-Sarkadi, & S. Tömöközi (Eds.), *Functional Foods. A New Challenge for the Food Chemist* (p. 109). Publishing Company of TUB. https://scholar.google.com/scholar_lookup?hl=en&publication_year=1999&pages=109&author=H.+Gylling&author=T.A.+Miettinen&title=Functional+Foods.+A+New+Challenge+for+the+Food+Chemist

Harborne, J. B. (Jeffrey B. ., Tomas-Barberan, F. A. (Francisco A. ., & Phytochemical Society of

Europe. (1991). *Ecological chemistry and biochemistry of plant terpenoids*. Clarendon Press. http://agris.fao.org/agris-search/search.do?recordID=US201300687965

Huang, Z.-R., Lin, Y.-K., & Fang, J.-Y. (2009). Biological and Pharmacological Activities of Squalene and Related Compounds: Potential Uses in Cosmetic Dermatology. *Molecules*, *14*, 540–554. https://doi.org/10.3390/molecules14010540

Lange, B. M., Wildung, M. R., McCaskill, D., & Croteau, R. (1998). A family of transketolases that directs isoprenoid biosynthesis via a mevalonate-independent pathway. *Proceedings of the National Academy of Sciences of the United States of America*, *95*(5), 2100–2104. https://doi.org/10.1073/pnas.95.5.2100

Laule, O., Fürholz, A., Chang, H.-S., Zhu, T., Wang, X., Heifetz, P. B., Gruissem, W., & Lange, M. (2003). Crosstalk between cytosolic and plastidial pathways of isoprenoid biosynthesis in Arabidopsis thaliana. *Proceedings of the National Academy of Sciences of the United States of America*, *100*(11), 6866–6871. https://doi.org/10.1073/pnas.1031755100

Liao, P., Hemmerlin, A., Bach, T. J., & Chye, M.-L. (2016). The potential of the mevalonate pathway for enhanced isoprenoid production. *Biotechnology Advances*, *34*(5), 697–713. https://doi.org/10.1016/J.BIOTECHADV.2016.03.005

Lois, L. M., Campos, N., Putra, S. R., Danielsen, K., Rohmer, M., & Boronat, A. (1998). Cloning and characterization of a gene from Escherichia coli encoding a transketolase-like enzyme that catalyzes the synthesis of D-1-deoxyxylulose 5-phosphate, a common precursor for isoprenoid, thiamin, and pyridoxol biosynthesis. *Proceedings of the National Academy of Sciences of the United States of America*, *95*(5), 2105–2110. https://doi.org/10.1073/pnas.95.5.2105

Mcgarvey, D. J., & Croteau, R. (1995). Terpenoid Metabolism. In *The Plant Cell* (Vol. 7). American Society of Plant Physiologists. https://www.ncbi.nlm.nih.gov/pmc/articles/PMC160903/pdf/071015.pdf

Römer, S., Fraser, P. D., Kiano, J. W., Shipton, C. A., Misawa, N., Schuch, W., & Bramley, P. M. (2000). Elevation of the provitamin A content of transgenic tomato plants. *Nature Biotechnology*, *18*(6), 666–669. https://doi.org/10.1038/76523

Rosati, C., Aquilani, R., Dharmapuri, S., Pallara, P., Marusic, C., Tavazza, R., Bouvier, F., Camara, B., & Giuliano, G. (2000). Metabolic engineering of beta-carotene and lycopene content in tomato fruit. *The Plant Journal*, *24*(3), 413–420. https://doi.org/10.1046/j.1365-313x.2000.00880.x

Savageau, M. A. (1971). Parameter sensitivity as a criterion for evaluating and comparing the performance of biochemical systems. *Nature*, *229*(5286), 542–544. https://doi.org/10.1038/229542A0

Savageau, M. A. (1975). Significance of autogenously regulated and constitutive synthesis of regulatory proteins in repressible biosynthetic systems. *Nature*, *258*(5532), 208–214. https://doi.org/10.1038/258208A0

Savageau, M. A. (1976). *Biochemical systems analysis : a study of function and design in molecular biology*. 379.

Schaller, H., Grausem, B., Benveniste, P., Chye, M. L., Tan, C. T., Song, Y. H., & Chua, N. H. (1995). Expression of the Hevea brasiliensis (H.B.K.) Mull. Arg. 3-Hydroxy-3-Methylglutaryl-Coenzyme A Reductase 1 in Tobacco Results in Sterol Overproduction. *Plant Physiology*, *109*(3), 761–770. https://doi.org/10.1104/pp.109.3.761

Schneider, M. M., Hampp, R., & Ziegler, H. (1977). Envelope Permeability to Possible Precursors of Carotenoid Biosynthesis during Chloroplast-Chromoplast Transformation. *Plant Physiology*, *60*(4), 518–520. https://doi.org/10.1104/pp.60.4.518

Sies, H., & Krinsky, N. I. (1995). The present status of antioxidant vitamins and beta-carotene. *The American Journal of Clinical Nutrition*, *62*(6), 1299S-1300S. https://doi.org/10.1093/ajcn/62.6.1299S

Sorribas, A., Hernández-Bermejo, B., Vilaprinyo, E., & Alves, R. (2007). Cooperativity and saturation in biochemical networks: a saturable formalism using Taylor series approximations. *Biotechnology and Bioengineering*, *97*(5), 1259–1277.

Voit, E. O. (1991). *Canonical nonlinear modeling : S-system approach to understanding complexity*. 365.

Voit, E. O. (2013). Biochemical Systems Theory: A Review. *ISRN Biomathematics*, *2013*, 1–53. https://doi.org/10.1155/2013/897658

Wołosik, K., Knaś, M., Zalewska, A., Niczyporuk, M., & Przystupa, A. W. (2013). The importance and perspective of plant-based squalene in cosmetology. *Journal of Cosmetic Science*, *64*(1), 59–66. http://www.ncbi.nlm.nih.gov/pubmed/23449131

World Health Organization. (2012). *WHO | Squalene-based adjuvants in vaccines*. World Health Organization. https://www.who.int/vaccine_safety/committee/topics/adjuvants/squalene/questions_and_answers/en/

Zorrilla-López, U., Masip, G., Arjó, G., Bai, C., Banakar, R., Bassie, L., Berman, J., Farré, G., Miralpeix, B., Pérez-Massot, E., Sabalza, M., Sanahuza, G., Vamvaka, E., Twyman, R. M., Christou, P., Zhu, C., & Capell, T. (2013). Engineering metabolic pathways in plants by multigene transformation. *The International Journal of Developmental Biology*, *57*(6-7–8), 565–576. https://doi.org/10.1387/ijdb.130162pc

Appendix A. Supplementary Figures

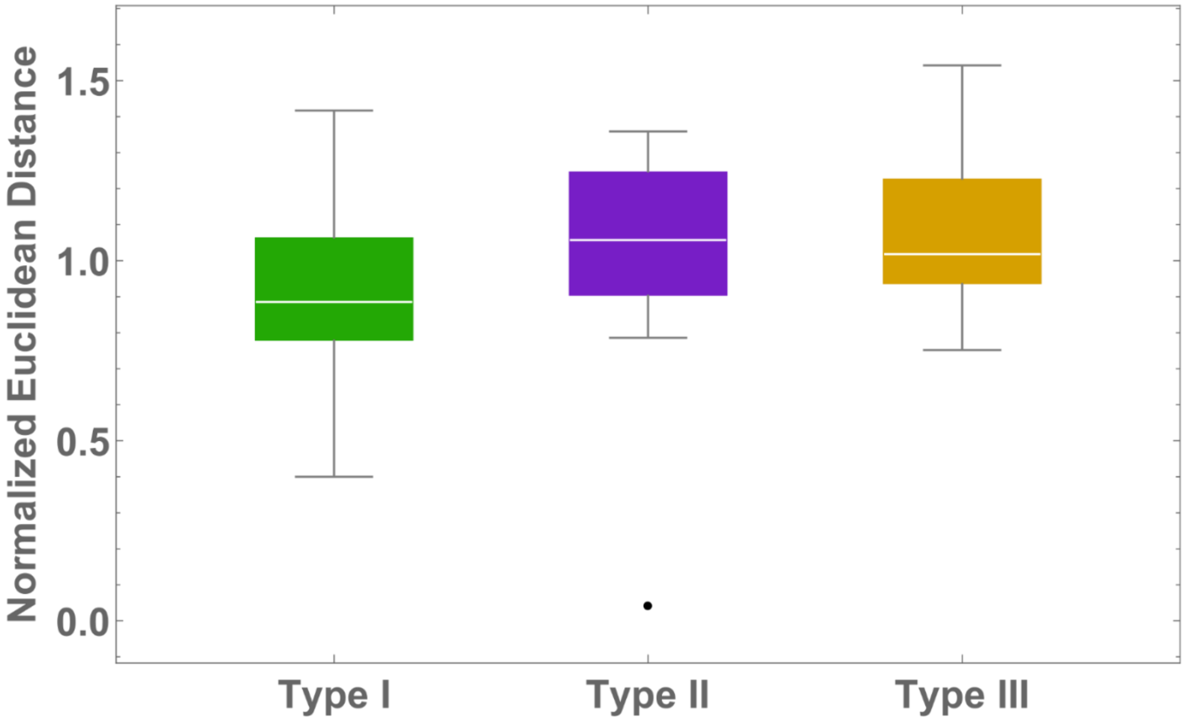

Supplementary Figure S1. Distribution of each line's Normalized Euclidean distance of Vmax9, Vmax12 and Vmax13 to the original values.

Appendix B. Supplementary tables.

Table S1. MVA pathway reactions that were considered in the model.

MVA pathway (cytoplasm)	Rate Expression	Rate
Acetoacetil-CoA_{cyt} → HMG-CoA_{cyt}	$(Vmax1\ HMGS\ acetoacetyl\text{-}CoA_{cyt})/(acetoacetyl\text{-}CoA_{cyt} + Km1\ (1 + (HMG\text{-}CoA_{cyt})/Ki1)\ )$	r_1
HMG-CoA_{cyt} → MVA_{cyt}	$\frac{Vmax2\ HMGR\ HMG\text{-}CoA_{cyt}}{HMG\text{-}CoA_{cyt} + Km2\left(1 + \frac{MVA_{cyt}}{Ki2}\right)}$	r_2
MVA_{cyt} → MVP_{cyt}	$\frac{Vmax3\ MVK\ MVA_{cyt}}{MVA_{cyt} + Km3\left(1 + \frac{MVP_{cyt}}{Ki31} + \frac{FPP_{cyt}}{Ki32} + \frac{GPP_{cyt}}{Ki33} + \frac{GGPP_{cyt}}{Ki34} + \frac{PhyPP_{cyt}}{Ki35}\right)}$	r_3
MVP_{cyt} → $MVPP_{cyt}$	$\frac{Vmax4\ PMK\ MVP_{cyt}}{MVP_{cyt} + Km4\left(1 + \frac{MVPP_{cyt}}{Ki4}\right)}$	r_4
$MVPP_{cyt}$ → IPP_{cyt}	$\frac{Vmax5\ MVD\ MVPP_{cyt}}{MVPP_{cyt} + Km5}$	r_5
IPP_{cyt} → $DMAPP_{cyt}$	$\frac{Vmax6\ IDI\ IPP_{cyt}}{IPP_{cyt} + Km6\left(1 + \frac{DMAPP_{cyt}}{Ki6}\right)}$	r_6
$DMAPP_{cyt}$ → IPP_{cyt}	$\frac{Vmax7\ IDI\ DMAPP_{cyt}}{DMAPP_{cyt} + Km7}$	r_7
2 IPP_{cyt} + 4 $DMAPP_{cyt}$ →	$k1\ IPP_{cyt} DMAPP_{cyt}$	r_{20}

Table S2. MEP pathway reactions that were considered in the model.

MEP pathway (plastid)	Rate Expressions	Rate
Glyceraldehyde-3-P + Pyruvate → DXP	$\frac{Vmax8\ DXS\ Pyruvate}{Pyruvate + Km8\left(1 + \frac{IPP_{pl}}{Ki81} + \frac{DMAPP_{pl}}{Ki82}\right)}$	r_{10}
DXP → MEP	$\frac{Vmax9\ DXR\ DXP_{pl}}{DXP_{pl} + Km9}$	r_{11}
MEP → CDP-ME	$\frac{Vmax10\ MCT\ MEP_{pl}}{MEP_{pl} + Km10}$	r_{12}
CDP-ME→CDP-MEP	$\frac{Vmax11\ CMK\ CDP - ME_{pl}}{CDP - ME_{pl} + Km11}$	r_{13}
CDP-MEP → MEcPP	$\frac{Vmax12\ MDS\ CDP - MEP_{pl}}{CDP - MEP_{pl} + Km12}$	r_{14}
MEcPP → HMBPP	$\frac{Vmax13\ HDS\ MEcPP_{pl}}{MEcPP_{pl} + Km13}$	r_{15}
HMBPP → IPP_{pl}	$\frac{Vmax14\ HDR\ HMBPP_{pl}}{HMBPP_{pl} + Km14}$	r_{16}
HMBPP → $DMAPP_{pl}$	$\frac{Vmax14\ HDR\ HMBPP_{pl}}{HMBPP_{pl} + Km14}$	r_{17}
IPP_{pl} → $DMAPP_{pl}$	$\frac{Vmax6\ IDI\ IPP_{\mathrm{pl}}}{IPP_{\mathrm{pl}} + Km6\left(1 + \frac{DMAPP_{\mathrm{pl}}}{Ki6}\right)}$	r_{18}
$DMAPP_{pl}$ → IPP_{pl}	$\frac{Vmax7\ IDI\ DMAPP_{\mathrm{pl}}}{DMAPP_{\mathrm{pl}} + Km7}$	r_{19}
3 IPP_{cyt} + $DMAPP_{cyt}$ →	$k1'\ IPP_{\mathrm{cyt}} DMAPP_{\mathrm{cyt}}$	r_{22}

Table S3. Exchange of MVA and MEP intermediates between the cytosol and the plastid.

Exchange of material between cytoplasm and plastid	Rate expressions	Rate
IPP_{cyt} → IPP_{pl}	$k2\ IPP_{cyt}$	r_8
IPP_{pl} → IPP_{cyt}	$k3\ IPP_{pl}$	r_9
$DMAPP_{cyt}$ → $DMAPP_{pl}$	$k2'\ DMAPP_{cyt}$	r_{25}
$DMAPP_{pl}$ → $DMAPP_{cyt}$	$k3'\ DMAPP_{pl}$	r_{26}
$HMG\text{-}CoA_{cyt}$ → $HMG\text{-}CoA_{pl}$	$k4\ HMG\text{-}CoA_{cyt}$	r_{32}
$HMG\text{-}CoA_{pl}$ → $HMG\text{-}CoA_{cyt}$	$k5\ HMG\text{-}CoA_{pl}$	r_{33}
MVA_{cyt} → MVA_{pl}	$k6\ MVA_{cyt}$	r_{34}
MVA_{pl} → MVA_{cyt}	$k7\ MVA_{pl}$	r_{35}
MVP_{cyt} → MVP_{pl}	$k8\ MVP_{cyt}$	r_{36}
MVP_{pl} → MVP_{cyt}	$k9\ MVP_{pl}$	r_{37}
$MVPP_{cyt}$ → $MVPP_{pl}$	$k10\ MVP_{cyt}$	r_{38}
$MVPP_{pl}$ → $MVPP_{cyt}$	$k11\ MVP_{pl}$	r_{39}

Table S4. Ectopic MVA pathway reactions that were considered in the model.

Ectopic MVA plastid pathway	Rate Expressions	Rate
Acetoacetil-CoA$_{pl}$ → HMG-CoA$_{pl}$	$(Vmax1\ HMGS_{Bj}\ acetoacetyl\text{-}CoA_{\mathrm{pl}})/(acetoacetyl\text{-}CoA_{\mathrm{pl}} + Km1\ (1 + (HMG\text{-}CoA_{\mathrm{pl}})/Ki1)\ )$	r_{27}
HMG-CoA$_{pl}$ → MVA$_{pl}$	$\frac{Vmax2\ tHMGR\ HMG\text{-}CoA_{\mathrm{pl}}}{HMG\text{-}CoA_{\mathrm{pl}} + Km2\ \left(1 + \frac{MVA_{\mathrm{pl}}}{Ki2}\right)}$	r_{28}
MVA$_{pl}$ → MVP$_{pl}$	$\frac{Vmax3\ crMVK\ MVA_{\mathrm{pl}}}{MVA_{\mathrm{pl}} + Km3\ \left(1 + \frac{MVP_{\mathrm{pl}}}{Ki31} + \frac{FPP_{\mathrm{pl}}}{Ki32} + \frac{GPP_{\mathrm{pl}}}{Ki33} + \frac{GGPP_{\mathrm{pl}}}{Ki34} + \frac{PhyPP_{\mathrm{pl}}}{Ki35}\right)}$	r_{29}
MVP$_{pl}$ → MVPP$_{pl}$	$\frac{Vmax4\ crPMK\ MVP_{\mathrm{pl}}}{MVP_{\mathrm{pl}} + Km4\ \left(1 + \frac{MVPP_{\mathrm{pl}}}{Ki4}\right)}$	r_{30}
MVPP$_{p}$ → IPP$_{pl}$	$\frac{Vmax5\ crMVD\ MVPP_{\mathrm{pl}}}{MVPP_{\mathrm{pl}} + Km5}$	r_{31}

Table S5. Kinetic Parameters. All concentration units in mM. All time units in s^{-1}.

Parameter	Value	Reference
Vmax1	0.454	Biochem J. 383:517-27
Km1	0.043	
Ki1	0.009	
Vmax2	0.033	Phytochemistry 21:2613-2618
Km2	0.056	J. Mol. Recognit. 21, 224-232
Ki2	0.081	Biochem. J. 381, 831-840
Vmax3	234.4	Int. J. Biol. Macromol. 72, 776-783<br>Biochem. J. 133, 335-347<br>Biochim. Biophys. Acta 279, 290-296<br>Org. Lett. 8, 1013-1016
Km3	0.046	
Ki31	0.18	
Ki32	0.0071	
Ki33	0.031	
Ki34	0.049	
Ki35	0.0036	
Vmax4	27.53	Phytochemistry 52, 975-983
Km4	0.35	Biochemistry 19, 2305-2310
Ki4	0.014	J. Biol. Chem. 278, 4510-4515
Vmax5	9.3	Phytochemistry 24, 2569-2571
Km5	0.01	Biochemistry 44, 2671-2677
Vmax6	5.7	Eur. J. Biochem. 249, 161-170
Km6	0.005	Eur. J. Biochem. 271, 1087-1093
Ki6	0.092	PNAS 108, 20461-20466
Vmax7	5.7	Eur. J. Biochem. 249, 161-170
Km7	0.017	
k1	2.0×10^{6}	Fitted
k1’	1.6×10^{4}	

Table S5 (continued). Kinetic Parameters. All concentration units in mM. All time units in s^{-1}.

Parameter	Value	Reference
Vmax8	1.22	J. Biol. Chem. 288, 16926-16936
Km8	0.019	
Ki81	0.065	
Ki82	0.081	
Vmax9	1.2	FEBS J. 273, 4446-4458
Km9	0.15	Plant Sci. 169, 287-294
Vmax10	31.17	Biochemistry 43, 12189-12197
Km10	0.37	
Vmax11	174.8	Chem Biol. 16:1230-1239
Km11	0.2	Bioorg. Med. Chem. 19, 5886-5895
Vmax12	0.61	ChemMedChem 5, 1092-1101
Km12	0.48	
Vmax13	0.20	J. Org. Chem. 70, 9168-9174
Km13	0.7	
Vmax14	4.18	J. Korean Soc. Appl. Biol. Chem. 56, 35-40
Km14	0.03	
k2, k2'	0.1	PNAS 100, 6866-6871<br>Arch Biochem Biophys. 415, 146-54
k3, k3'	1	PNAS 100, 6866-6871<br>Arch Biochem Biophys. 415, 146-54
k4, k6, k8, k10	1000	Assumes rapid equilibrium between cytoplasm and plastid, in the absence of quantitative information about exchange. Equilibrium is favored towards the cytoplasm<br>PNAS 100, 6866-6871
k5, k7, k9, k11	10000	

Table S6. Concentration of independent Variables (Albe et al., 1990).

Metabolite	*Concentration (mM)*
Ac-CoA	0.350
GA3P	0.006
Pyruvate	1.600

Table S7. Expressions for each hormone effect.

	Effect	Reaction it is applied to
**Mutant Type I**	$iP^{0.44}$	r_3
	$IAA^{-0.26}$	r_4
	$IAA^{0.28}$	
	$\left(\frac{iP}{0.069 + iP}\right)^{0.58}$	r_{13}
	$\left(\frac{ABA}{0.023 + ABA}\right)^{0.98}$	
	$iP^{0.36}$	r_6, r_7, r_{18}, r_{19}
	$IAA^{0.14}$	r_{28}
**Mutant Type II**	$iP^{0.18}$	r_{18}, r_{22}
	$iP^{0.19}$	r_{19}, r_{22}
	$GA3^{-0.32}$	r_2
	$\left(\frac{tZ}{0.011 + tZ}\right)^{-2.27}$	r_3
	$GA4^{0.25}$	r_4
	$\left(\frac{tZ}{0.031 + tZ}\right)^{-2.26}$	r_5
	$GA4^{0.26}$	
	$GA4^{0.33}$	r_{13}
	$GA4^{0.26}$	
	$JA^{-1.06}$	

	${\frac{ZR}{61.6+ZR}}^{0.89}$	r_{16}, r_{17}
	${\frac{IAA}{9.99\cdot 10^3+IAA}}^{0.58}$	
	${\frac{JA}{0.351+JA}}^{1.11}$	
	$ZR^{0.41}$	r_6, r_7, r_{18}, r_{19}
	$GA3^{-1.07}$	r_{11}
	$IAA^{-0.91}$	
	${\frac{ABA}{0.005+ABA}}^{2.44}$	
	$GA4^{0.34}$	r_{14}
**Mutant Type III**	$GA1^{0.27}$	r_{17}, r_{18}
	${\frac{GA3}{17.5+GA3}}^{1.15}$	r_1
	${\frac{ZR}{1.20+ZR}}^{-1.09}$	r_2
	$ZR^{-1.31}$	r_3
	${\frac{GA3}{0.200+GA3}}^{-1.76}$	r_4
	${\frac{GA3}{0.329+GA3}}^{-2.46}$	r_5
	${\frac{GA1}{0.813+GA1}}^{1.20}$	r_{10}
	$ACC^{-0.52}$	r_{12}

	$\left(\frac{iP}{1.08 + iP}\right)^{5.70}$	r_{13}
	$GA3^{-1.07}$	
	$IAA^{0.25}$	r_{16}, r_{17}
	$IAA^{1.27}$	
	$\left(\frac{iP}{0.344 + iP}\right)^{8.22}$	
	$\left(\frac{GA3}{0.014 + GA3}\right)^{-1.02}$	
	$\left(\frac{ABA}{0.004 + ABA}\right)^{-2.62}$	r_6, r_7, r_{18}, r_{19}
	$GA3^{0.5}$	
	$\left(\frac{ZR}{0.145 + ZR}\right)^{1.09}$	r_{27}
	$\left(\frac{GA3}{2.37 + GA3}\right)^{1.15}$	
	$\left(\frac{GA3}{0.059 + GA3}\right)^{1.78}$	r_{29}
	$\left(\frac{GA3}{0.303 + GA3}\right)^{2.98}$	r_{30}
	$GA3^{0.35}$	r_{11}
	$ZR^{0.90}$	
	$\left(\frac{ABA}{0.021 + ABA}\right)^{1.56}$	r_{14}
	$IAA^{0.43}$	
	$GA3^{0.35}$	r_{15}

$$\frac{ABA^{0.84}}{\frac{tZ}{0.003 + tZ}^{-2.86}}$$

Table S8. Logarithmic sensitivities of the eigenvalues to reaction parameters. Only the top 24 sensitivities with largest absolute value are shown. Full table in Supplementary Data S1.

Eigenvalue	Parameter	Logarithmic sensitivity	Eigenvalue	Parameter	Logarithmic sensitivity
EV5	Km3	-13965	EV5	Ki81	-4881
EV5	Ki31	-13961	EV5	Ki82	-4374
EV5	Km4	-13951	EV5	$Km6_{pl}$	-1813
EV5	Ki4	-13933	EV5	k1	1745
EV5	Ki34	-13468	EV13	Vmax4	1024
EV5	Ki33	-13178	EV12	Vmax3	1009
EV5	$Ki6_{pl}$	-12927	EV6	Km3	-221
EV5	Ki32	-10525	EV6	Ki31	-221
EV5	Ki2	-10475	EV6	Km4	-221
EV5	Ki6	-9495	EV6	Ki4	-220
EV5	Km6	-7504	EV6	Ki34	-213
EV5	Ki35	-7179	EV6	Ki33	-208

Table S9. Logarithmic sensitivities of the concentrations to reaction parameters. Only sensitivities with an absolute value larger than 0.5 are shown.

Variable	Parameter	Sensitivity
$HMGCoA_{cyt}$	Ki1	0.945
$HMGCoA_{cyt}$	Km1	-0.953
$HMGCoA_{cyt}$	Vmax1	1.02
$HMGCoA_{cyt}$	Vmax2	-1.02
MVA_{cyt}	Vmax2	0.945
MVA_{cyt}	Km3	1.00
MVA_{cyt}	Vmax3	-1.00
MVP_{cyt}	Vmax2	0.948
MVP_{cyt}	Km4	1.00
MVP_{cyt}	Vmax4	-1.00
$MVPP_{cyt}$	Vmax2	0.948
$MVPP_{cyt}$	Km5	1.00
$MVPP_{cyt}$	Vmax5	-1.00
IPP_{cyt}	Vmax6	-0.929
IPP_{cyt}	Vmax7	1.79
IPP_{pla}	$Vmax6_{pl}$	-2.86
IPP_{pla}	$Vmax7_{pl}$	2.89
IPP_{pla}	k1’	-0.621
$DMAPP_{cyt}$	Vmax6	0.929
$DMAPP_{cyt}$	Vmax7	-1.79
$DMAPP_{cyt}$	k1’	-0.500
$DMAPP_{pla}$	$Vmax6_{pl}$	2.86
$DMAPP_{pla}$	$Vmax7_{pl}$	-2.89
DXP	Km8	-1.00
DXP	Vmax8	1.09
DXP	Km9	1.00
DXP	Vmax9	-1.09

Variable	Parameter	Sensitivity
MEP	Km8	-0.922
MEP	Vmax8	1.00
MEP	Km10	1.00
MEP	Vmax10	-1.00
CDPME	Km8	-0.920
CDPME	Vmax8	0.999
CDPME	Km11	1.00
CDPME	Vmax11	-1.00
CDPMEP	Km8	-1.09
CDPMEP	Vmax8	1.19
CDPMEP	Km12	1.00
CDPMEP	Vmax12	-1.19
MECPP	Ki81	0.584
MECPP	Ki82	0.725
MECPP	Km8	-1.79
MECPP	Vmax8	1.94
MECPP	Km13	1.00
MECPP	Vmax13	-1.94
MECPP	k1’	0.638
HMBPP	Km8	-0.930
HMBPP	Vmax8	1.01
HMBPP	Vmax14	-0.505
HMBPP	Km14	0.501
HMBPP	Vmax14’	-0.507

Table S10. Levels of each intermediary metabolite (left) at the steady states of each mutant type's average model and the eigenvalues (right) of the Jacobian matrix at these steady states.

Steady state concentrations [mM]

	Type I	Type II	Type III
**$HMGCoA_{cyt}$**	5.76×10^{-1}	4.67	2.97
**MVA_{cyt}**	4.86×10^{-5}	1.15×10^{-4}	6.55×10^{-5}
**MVP_{cyt}**	6.61×10^{-4}	1.57×10^{-3}	8.43×10^{-4}
**$MVPP_{cyt}$**	9.68×10^{-5}	1.41×10^{-4}	7.53×10^{-5}
**IPP_{cyt}**	1.14×10^{-3}	9.42×10^{-4}	7.93×10^{-4}
**IPP_{pla}**	7.45×10^{-4}	9.22×10^{-4}	1.09×10^{-3}
**$DMAPP_{cyt}$**	8.19×10^{-6}	1.35×10^{-5}	1.58×10^{-5}
**$DMAPP_{pla}$**	2.90×10^{-3}	3.41×10^{-3}	3.96×10^{-3}
**DXP**	5.24×10^{-1}	8.41×10^{-1}	1.06
**MEP**	4.58×10^{-3}	7.10×10^{-3}	5.10×10^{-3}
**CDPME**	4.12×10^{-4}	3.45×10^{-4}	4.60×10^{-4}
**CDPMEP**	7.55×10^{-1}	2.76	3.48
**MECPP**	9.46	4.00	5.05
**HMBPP**	1.08×10^{-3}	1.73×10^{-3}	3.52×10^{-3}
**$HMGCoA_{pla}$**	5.76×10^{-2}	4.67×10^{-1}	2.97×10^{-1}
**MVA_{pla}**	9.37×10^{-6}	1.77×10^{-5}	1.10×10^{-5}
**MVP_{pla}**	-	1.60×10^{-4}	8.52×10^{-5}
**$MVPP_{pla}$**	-	-	8.86×10^{-6}

Stability

	Type I	Type II	Type III
**EV1**	-11249	-12515	-14205
**EV2**	-11000	-11009	-12624
**EV3**	-4678	-11000	-11306
**EV4**	-1994	-3946	-11000
**EV5**	-1124	-1098	-3373
**EV6**	-402	-1053	-2001
**EV7**	-357	-618	-1904
**EV8**	-163	-595	-669
**EV9**	-132	-156	-602
**EV10**	-91	-117	-184
**EV11**	-65	-85	-171
**EV12**	-32	-84	-98
**EV13**	-1.94×10^{-1}	-30	-71
**EV14**	-7.64×10^{-2}	-3.79×10^{-2}	-54
**EV15**	-6.17×10^{-2}	-2.70×10^{-2}	-4.42×10^{-2}
**EV16**	-1.09×10^{-3}	-1.04×10^{-2}	-3.20×10^{-2}
**EV17**	-	-8.93×10^{-3}	-8.61×10^{-3}
**EV18**	-	-	-7.70×10^{-3}

Table S11. Cross-validation of the models, showing the confidence intervals (95%) of the adjusted R^2 and relative errors, also when using a model to predict different mutant types.

	Adjusted R^2 (Mean [CI (95%)])	Log_{10} Relative error (Mean [CI (95%)])		
		Type I	Type II	Type III
**Chlorophyll I**	0.74 [0.72, 0.75]	-1.45 [-1.5, -1.41]	-0.71 [-0.72, -0.71]	-0.8 [-0.82, -0.78]
**Chlorophyll II**	0.72 [0.69, 0.75]	-0.62 [-0.63, -0.61]	-1.84 [-1.91, -1.77]	-0.87 [-0.88, -0.85]
**Chlorophyll III**	0.44 [0.38, 0.49]	-0.9 [-0.91, -0.89]	-1.21 [-1.23, -1.19]	-1.28 [-1.33, -1.23]
**Height I**	0.51 [0.47, 0.54]	-1.14 [-1.2, -1.08]	0.21 [0.12, 0.31]	-0.42 [-0.43, -0.42]
**Height II**	-	-	-	-
**Height III**	0.61 [0.57, 0.64]	0.25 [0.21, 0.29]	0.09 [0.03, 0.14]	-1.07 [-1.27, -0.87]
**Leaf Length I**	0.67 [0.64, 0.69]	-1.21 [-1.26, -1.15]	4.51 [4.47, 4.55]	-0.01 [-0.07, 0.05]
**Leaf Length II**	0.78 [0.75, 0.8]	-0.29 [-0.31, -0.27]	-1.09 [-1.22, -0.97]	-0.68 [-0.71, -0.65]
**Leaf Length III**	0.41 [0.37, 0.45]	-0.17 [-0.18, -0.16]	-0.71 [-0.73, -0.68]	-1.38 [-1.42, -1.33]
**Leaves I**	0.68 [0.63, 0.72]	-1.31 [-1.48, -1.15]	0.76 [0.66, 0.86]	-0.98 [-1.01, -0.96]
**Leaves II**	0.69 [0.64, 0.73]	-0.66 [-0.69, -0.63]	-1.5 [-1.73, -1.26]	-0.62 [-0.67, -0.58]
**Leaves III**	-	-	-	-
**Leaf Width I**	-	-	-	-
**Leaf Width II**	0.58 [0.54, 0.61]	-0.25 [-0.29, -0.22]	-0.95 [-1, -0.9]	-0.73 [-0.75, -0.71]
**Leaf Width III**	0.41 [0.39, 0.42]	-0.2 [-0.22, -0.19]	-0.62 [-0.65, -0.6]	-1.25 [-1.31, -1.19]
